# Effects of polystyrene microplastics on the growth and metabolism of highland barley seedlings based on LC-MS

**DOI:** 10.3389/fpls.2024.1477605

**Published:** 2024-12-17

**Authors:** Wenqi Xiao, Peng Xiang, Wenlong Liao, Zhuang Xiong, Lianxin Peng, Liang Zou, Bingliang Liu, Qiang Li

**Affiliations:** Key Laboratory of Coarse Cereal Processing, Ministry of Agriculture and Rural Affairs, Sichuan Engineering and Technology Research Center of Coarse Cereal Industrialization, School of Food and Biological Engineering, Chengdu University, Chengdu, Sichuan, China

**Keywords:** microplastics, highland barley, metabolomics, oxidative stress, metabolic pathways

## Abstract

Microplastics are widely present in the environment and can adversely affect plants. In this paper, the effects of different concentrations of microplastics on physiological indices and metabolites of highland barley were investigated for the first time using a metabolomics approach, and revealed the response mechanism of barley seedlings to polystyrene microplastics (PS-MPs) was revealed. The results showed that the aboveground biomass of highland barley exposed to low (10 mg/L) and medium (50 mg/L) concentrations of PS-MPs increased by 32.2% and 48.2%, respectively. The root length also increased by 16.4% and 21.6%, respectively. However, the aboveground biomass of highland barley exposed to high (100 mg/L) concentrations of PS-MPs decreased by 34.8%, leaf length by 20.7%, and root length by 25.9%. Microplastic exposure increased the levels of antioxidant activity, suggesting that highland barley responds to microplastic stress through oxidative stress. Metabolome analysis revealed that the contents of 4 metabolites increased significantly with increasing PS-MPs concentration in positive ionmode, while the contents of 8 metabolites increased significantly with increasing PS-MPs concentration in negative ionmode (*P* < 0.05), including prunin, dactylorhin E, and schisantherin B. Additionally, PS-MPs significantly interfered with highland barley flavonoid biosynthesis, pyrimidine metabolism, purine metabolism, fatty acid biosynthesis, and phenylpropanoid biosynthesis metabolic pathways. This study provides a new theoretical basis for a deeper understanding of the effects of different concentrations of PS-MPs on highland barley.

## 1 Introduction

Microplastics (MPs) are widespread in the environment and may adversely affect plants. Microplastics are fragments and particles less than 5 millimeters in diameter that result from physical, chemical, biological, and other forms of abrasion, consumption, and decomposition of larger plastics (Catarino et al., 2018; Napper et al., 2020; Xu et al., 2020). MPs are considered to be more serious and persistent pollutants than plastics. According to reports, 79% of the world’s plastic waste ends up in landfills (Horton et al., 2017; Rillig et al., 2017). Therefore, the soil could be a significant sink for MPs (Geyer et al., 2017). Agricultural film residues and sewage irrigation are important sources of MPs in soil, which can cause serious harm to plants (Steinmetz et al., 2016; Liu et al., 2018; Ng et al., 2018; Serrano-Ruiz et al., 2021). MPs can enter plants through the plant root system and adversely affect plant characteristics, growth, and nutrient uptake (Wan et al., 2019). Therefore, considering the hazards of MPs, there is a need to explore their uptake, accumulation, and transport in plants (Li et al., 2020a).

Polystyrene (PS) is widely used worldwide. The material is often used in the manufacture of products such as disposable tableware, insulation foam, and packaging for various goods. However, under natural environmental conditions, PS has a relatively long decomposition cycle, making it one of the most abundant microplastics in agroecosystems (Sun et al., 2020). However, current research on the effects of polystyrene microplastics (PS-MPs) on plants is limited. Therefore, it is urgent to elucidate the effects of PS-MPs on plants. Zhou et al (Zhou et al., 2021). demonstrated the possibility of transporting polystyrene nanoplastics (PS-NPs) in rice roots, and the significant enhancement of antioxidant enzyme activities reflected the oxidative stress response of rice roots to PS-NPs. Lian et al (Lian et al., 2021). found that MPs affected the synthesis and photosynthesis of lettuce chlorophyll, resulting in a reduction of micronutrients and ultimately affecting the yield. Similar phenomena were observed in lettuce by Gao et al (Gao et al., 2021a, Gao et al., 2021b). In addition, Hernandez-Arenas et al (Hernandez-Arenas et al., 2021). found that MPs also affected the yield of tomatoes.

Highland barley is the smallest staple and largest coarse grain species in China, and the main food crop for people living at high altitudes (Guo et al., 2020). It has been shown to have a range of dietary benefits, including being low in fat and sugar, high in fiber, and especially high in levels of beta-glucan (Zhou and Wu, 2022). Studies have shown that mulching with plastic film can improve the chlorophyll content, photosynthetic rate, and yield of highland barley. However, mulching with plastic film can also lead to a large amount of MPs remaining in the cultivated soil of highland barley, thus increasing the harm of highland barley exposure to MPs. To date, no study has comprehensively revealed the risk of exposure to MPs and the mechanisms of their response to MPs in highland barley.

Metabolomics is widely used to analyze the metabolic state and metabolic pathways of biological systems. The technique is capable of qualitatively and quantitatively analyzing all metabolites in a given biological sample under specific conditions (Fiehn et al., 2000). It has proven to be an effective tool for understanding changes in the chemical composition of crops and for further revealing the mechanism of this process (Soria et al., 2017). The remodeling of the metabolome under stress largely reflects the response and defense of plants to stress, and metabolomics technology provides a reliable means to study the remodeling of metabolites under different stresses. Metabolites play a variety of important roles in plant physiology, including providing energy and carbon sources, storing nutrients, synthesizing hormones, providing antioxidant protection, and enhancing stress tolerance. These roles are important for plant growth, development, and adaptation to the environment. At present, some studies have used metabolomics to analyze the changes in plant metabolites exposed to MPs, including rice (Wu et al., 2020a), lettuce (Wang et al., 2022a, Wang et al., 2023a), and Glycine max L (Qiu et al., 2023). Analysis of crop metabolite changes reveals that exposure to MPs triggers a biological detoxification mechanism to protect plants from this stress (Zhang et al., 2019). However, studies using metabolomics to assess the effects of MPs on crops are still lacking.

In this study, the effects of different concentrations of PS-MPs on physiological indicators and metabolites of plateau barley seedlings were investigated in depth for the first time using metabolomics techniques. This study provides valuable data and insights into the processes and mechanisms of potential toxicity of PS-MPs to highland barley and offers a new perspective on the effects of microplastics on plant production systems. This will help researchers to more comprehensively assess the potential hazards of microplastics.

## 2 Materials and methods

### 2.1 Experimental material

Non-fluorescent PS (particle size of 96 nm, density of 1.05 g/cm^3^) has a distribution coefficient of ≤ 5.8%. The red fluorescent-labeled polymethyl methacrylate (PMMA) (particle size of 103 nm, density of 1.18 g/cm^3^) has a distribution coefficient of ≤ 6.5%. PS was used to study the effects on the physiological and biochemical indicators of highland barley, and a red fluorescently labeled PMMA was used to examine the distribution of MPs in the roots of highland barley seedlings. The fluorescence excitation and emission wavelengths of the fluorescently labeled PMMA were 630 nm and 680 nm, respectively. Highland barley seeds (white barley) were purchased from Orson Horticultural Company (Jiangsu, China). PS-MPs were dispersed in a Hoagland nutrient solution using a DTD series sonicator (SB-800DTD, China) to make the PS-MPs concentrations reach 10 mg/L, 50 mg/L, and 100 mg/L. The concentrations of PS-MPs were determined based on phytotoxicological test exposure concentrations (Besseling et al., 2014; Leifheit et al., 2021).

### 2.2 Experimental design

Highland barley seeds were surface sterilized with a 2% NaClO solution for 30 minutes, washed with distilled water 3 times, and germinated in a seedling tray filled with purified water at 25°C in the dark for 2 days. Subsequently, seedlings with consistent germination were selected and transplanted into 1/2 strength Hoagland solution (Shorobi et al., 2023) (1 mM KNO_3_, 1 mM Ca(NO_3_)_2_·4H_2_O, 4 mM MgSO_4_·7H_2_O, 0.2 mM NH_4_NO_3_, 0.1 mM KH_2_PO_4_, iron solution (4 μM Fe-EDTA), and micronutrients (9.2 μM H_3_BO_3_, 1.8 μM MnCl_2_·4H_2_O, 0.15 μM ZnSO_4_·7H_2_O, 0.04 μM CuSO_4_·5H_2_O and 0.1 μM H_2_MoO_4_·H_2_O), with a pH of 5.5). The seedlings were placed in seedling trays, each containing 25 seedlings (5 columns x 5 rows). Each seedling tray was filled with 700 mL of Hoagland’s solution. The control group was grown in Hoagland’s solution without PS-MPs. In addition, 9 germinated seeds were selected to grow in nursery trays containing fluorescently labeled PMMA at a concentration of 0.5 g/L in Hoagland’s solution (100 mL) to examine the distribution of PMMA. The solutions above were changed every six days. There were six replicates per treatment group. All seedling trays were placed in an artificial climate chamber (RXZ-300B, Kaled Technology Co., Ltd., China) with 16 hours of light during the day, a light intensity of 6,000 lx, and a temperature of 25°C; 8 hours at night, the temperature was 20°C, and the relative humidity was 60%. After 12 days of treatment, leaf and root samples were collected to determine physiological indicators and enzyme activities. For metabolic analysis, we selected root samples from three treatment groups: control, low concentration (10 mg/L), and high concentration (100 mg/L), with six replicates for each treatment group, for a total of 18 samples.

### 2.3 Determination of growth indices of highland barley seedlings

At the end of the test, the physical properties of seedlings in each group (25 samples in each group) were recorded, including root length (cm), root fresh weight (g), leaf length (cm), and leaf fresh weight (g). A root scanner (WinRHIZO, Canada) was used to acquire high-resolution images of barley seedlings in the highlands. The experiments were performed in six replicates.

### 2.4 Detection of PMMA absorption in highland barley roots by confocal scanning microscopy

Confocal Laser Scanning Microscopy (CLSM, Olympus FV10i, Japan) was used to detect the distribution of fluorescently labeled PMMA in the roots. Before the commencement of the test, the instrument was accurately calibrated and verified. Rigorous tests were conducted using standard samples to ensure the stability of the instrument’s performance, as well as the accuracy and reliability of the results obtained. All root samples were sonicated for 5 minutes and washed three times with distilled water to remove any possible residual PMMA attached to the surface. According to Kuixian et al (Kuixian et al., 2010), a 1 cm root tip was cut with a stainless steel blade and fixed in 0.5% glutaraldehyde for 30 min to enhance fluorescence intensity. Scan the z-plane of the root, where the central vascular bundle serves as the internal reference. Confocal laser scanning was repeated at least three times before selecting representative images. The excitation and emission wavelengths were 630 nm and 680 nm, respectively.

### 2.5 Biochemical analysis of highland barley seedlings

To assess the possible physiological and biochemical effects of PS-MPs on highland barley seedlings, root and leaf samples of uniformly growing seedlings were selected from each treatment group after 12 days of treatment. Root and leaf samples were collected using clean tools to ensure that the samples were not contaminated. The samples were rinsed three times with ultrapure water after collection to avoid adsorption of PS-MPs. After the sample collection was completed, the samples were stored in a -80°C refrigerator. Next, catalase (CAT), peroxidase (POD), superoxide dismutase (SOD), malondialdehyde (MDA), superoxide anion (O_2_^-^), and hydrogen peroxide were quantitatively determined using a commercially available assay kit (supplied by Nanjing Jianjian Biological Co., Ltd., China). The specific steps of the experiment include sample pretreatment, extraction, centrifugation, and finally, the data is analyzed using a multifunctional microplate reader. The results are processed and interpreted according to the relevant calculation formula. In the sample pretreatment stage, the sample needs to be fully dissolved and diluted first to make it easier to analyze. In the extraction stage, specific extraction reagents are used to separate the substances to be analyzed from the sample. This facilitates subsequent analyses. The centrifugation step is used to remove impurities and unwanted substances from the sample, improving the accuracy of the analysis. Each sample is repeated six times to ensure the stability and reliability of the experimental data.

### 2.6 Analysis of metabolites in highland barley roots

For the Liquid Chromatography-Mass Spectrometry (LC-MS) nontargeted metabolomic analysis of highland barley root metabolites (Want et al., 2010; Dunn et al., 2011), the experimental process mainly included sample metabolite extraction, LC-MS detection, and data analysis. Metabolome analysis was performed on 6 replicates with a sample size of >200 mg (fresh weight) per replicate. First, the plant root samples were frozen and ground into powder form; then, an 80% aqueous methanol solution was added and subjected to vortex shaking, followed by extraction in an ice-water bath for 1 h; Immediately thereafter, the metabolites were extracted by centrifugation (15,000g, 4°C for 20 min); finally, the metabolites in the extracts were analyzed using LC-MS for qualitative and quantitative analysis (Gromova and Roby, 2010; Hiraga et al., 2020). We also performed quality control (QC) to ensure the stability of the system. The raw data from the machine was preprocessed using the CD3.1 data processing software. First, simply screen through parameters such as retention time and mass-to-charge ratio. For each sample, peak alignment was performed based on retention time deviation, mass deviation (parts per million, ppm), signal-to-noise ratio (S/N), adduct ions, and other information to ensure accurate identification. Peaks were extracted and their corresponding peak areas were quantified simultaneously. Then, the high-resolution secondary spectrum databases mzCloud and mzVault and the MassList primary database were searched to identify metabolites. Next, the metabolites were subjected to multivariate statistical analysis, including principal component analysis (PCA) and partial least squares discriminant analysis (PLS-DA), to reveal differences in metabolic patterns among different groups. Hierarchical clustering (HCA) and metabolite correlation analysis were used to reveal relationships between samples and among metabolites. Finally, the biological significance associated with metabolites is explained through functional analysis, such as metabolic pathways.

### 2.7 Statistical analysis

In this study, three replicated experiments (n = 3) were used for analysis, while metabolites were assayed in six plant samples treated under the same conditions. The data for this study were analyzed using analysis of variance (ANOVA) with SPSS 21.0 statistical software. The data is expressed as the mean ± standard error. For comparisons between groups, a p-value of less than 0.05 was considered a significant difference. Multivariate statistical analysis will use an unsupervised principal component analysis (PCA) to observe the overall distribution among samples and the stability of the overall analysis process. Statistical analysis of significant differences was assessed using a one-way analysis of variance (ANOVA) and Tukey’s honestly significant difference (HSD) test.

## 3 Results

### 3.1 Effects of PS-MPs on biomass and length of barley seedlings

**Figure 1:**
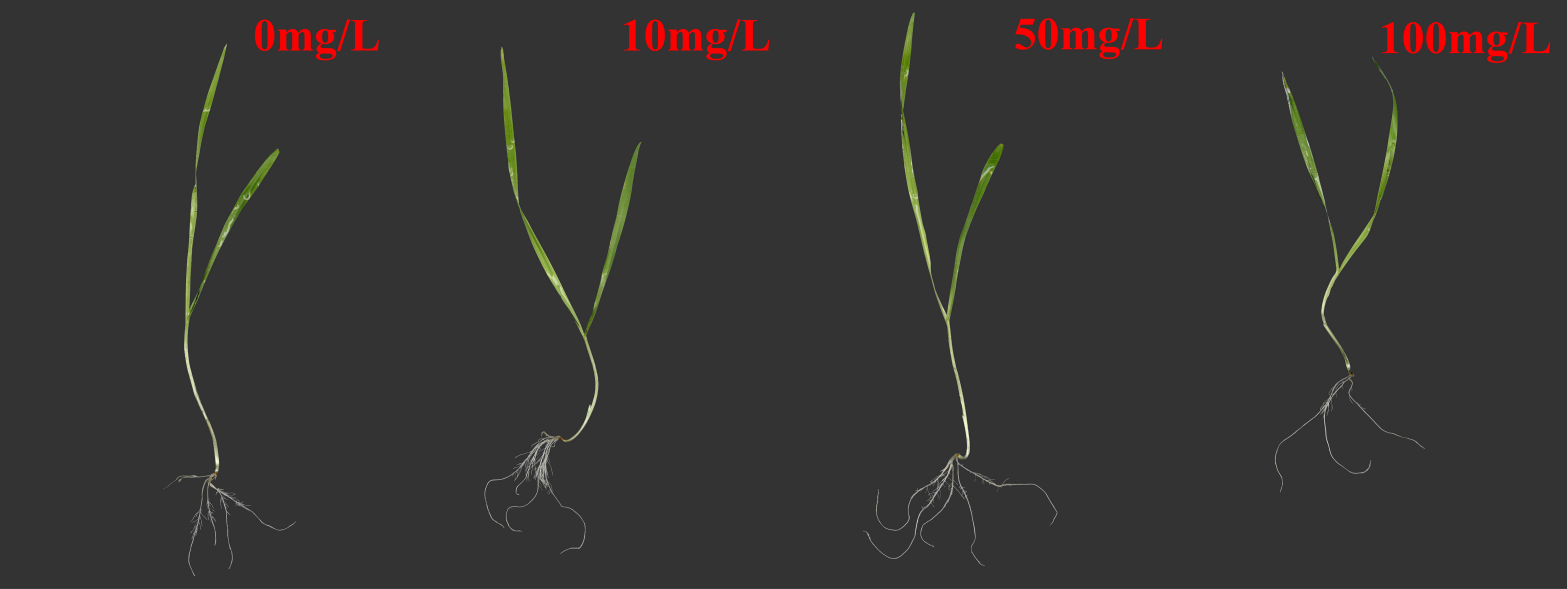
Morphology of highland barley seedlings after 12 days of exposure to different concentrations of PS-MPs.

**Figure 2:**
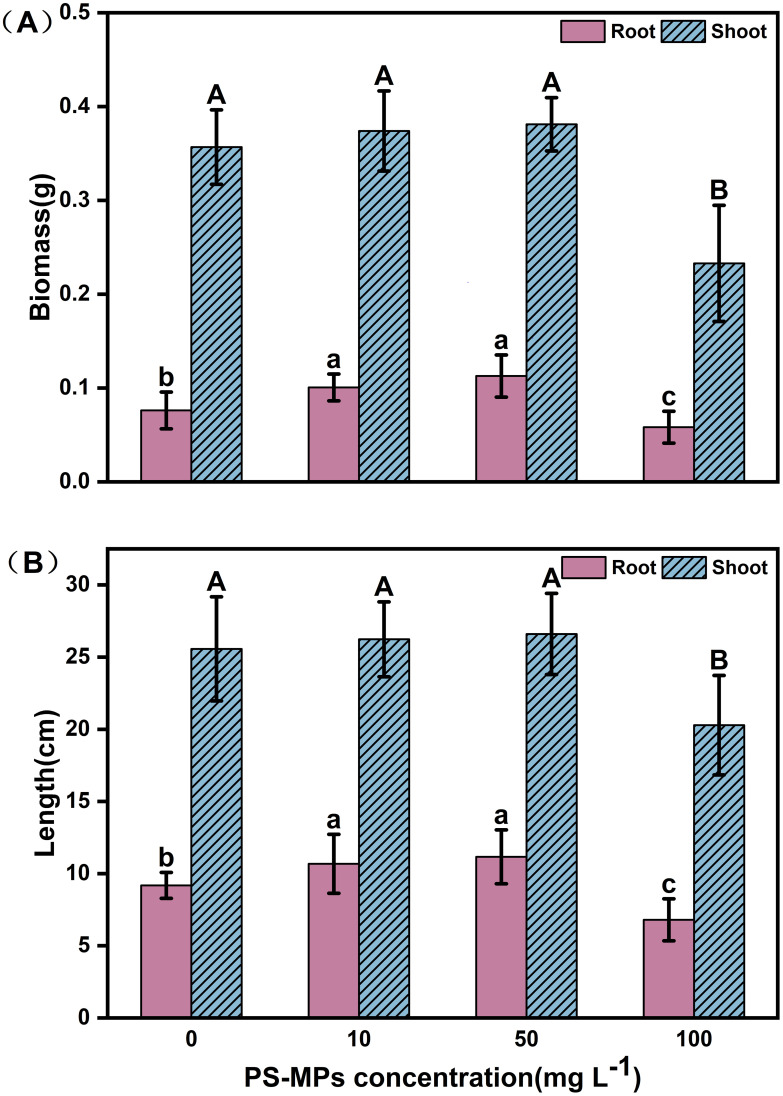
Biomass and length of highland barley roots and leaves after 12 days of exposure to PS-MPs. **(A)** biomass of roots and leaves **(B)** lengths of roots and leaves. (Upper-case letters represent differences between leaf samples, and lower-case letters represent differences between root samples. Different letters indicate significant differences between samples) (*P* < 0.05).

Microplastics have a significant effect on plant growth. In this study, **Figure 1** shows the specific biomass, root length, and leaf length of plateau barley seedlings after 12 days of exposure to PS-MPs. It can be seen from the figure that low concentrations (10 mg/L) and medium concentrations (50 mg/L) treatments promoted the growth of highland barley seedlings, while a high concentration (100 mg/L) treatment inhibited the growth of highland barley seedlings. As shown in **Figure 2A**, the below-ground biomass of highland barley exposed to low (10 mg/L) and medium (50 mg/L) concentrations of PS-MPs increased by 32.2% and 48.2%, respectively, which was significantly higher than that of the control group (*P* < 0.05). Meanwhile, the underground biomass exposed to high concentrations of PS-MPs decreased by 23.5%, which was significantly lower than that of the control group (*P* < 0.05). In addition, exposure to low and medium concentrations of PS-MPs did not have a significant effect on aboveground biomass. However, exposure to high concentrations of PS-MPs resulted in a 34.8% decrease in aboveground biomass, which was significantly lower than that of the control group (*P* < 0.05). Similar effects were also observed for the lengths of the roots and leaves of highland barley seedlings (**Figure 2B**). The root lengths of PS-MPs exposed to low (10 mg/L) and medium (50 mg/L) concentrations increased by 16.4% and 21.6%, respectively, which were significantly higher than those of the control group (*P* < 0.05). However, the root and leaf lengths exposed to high concentrations of PS-MPs were reduced by 25.9% and 20.7%, respectively. These values were significantly lower than those of the control group (*P* < 0.05).

### 3.2 Uptake of PMMA by highland barley roots

**Figure 3:**
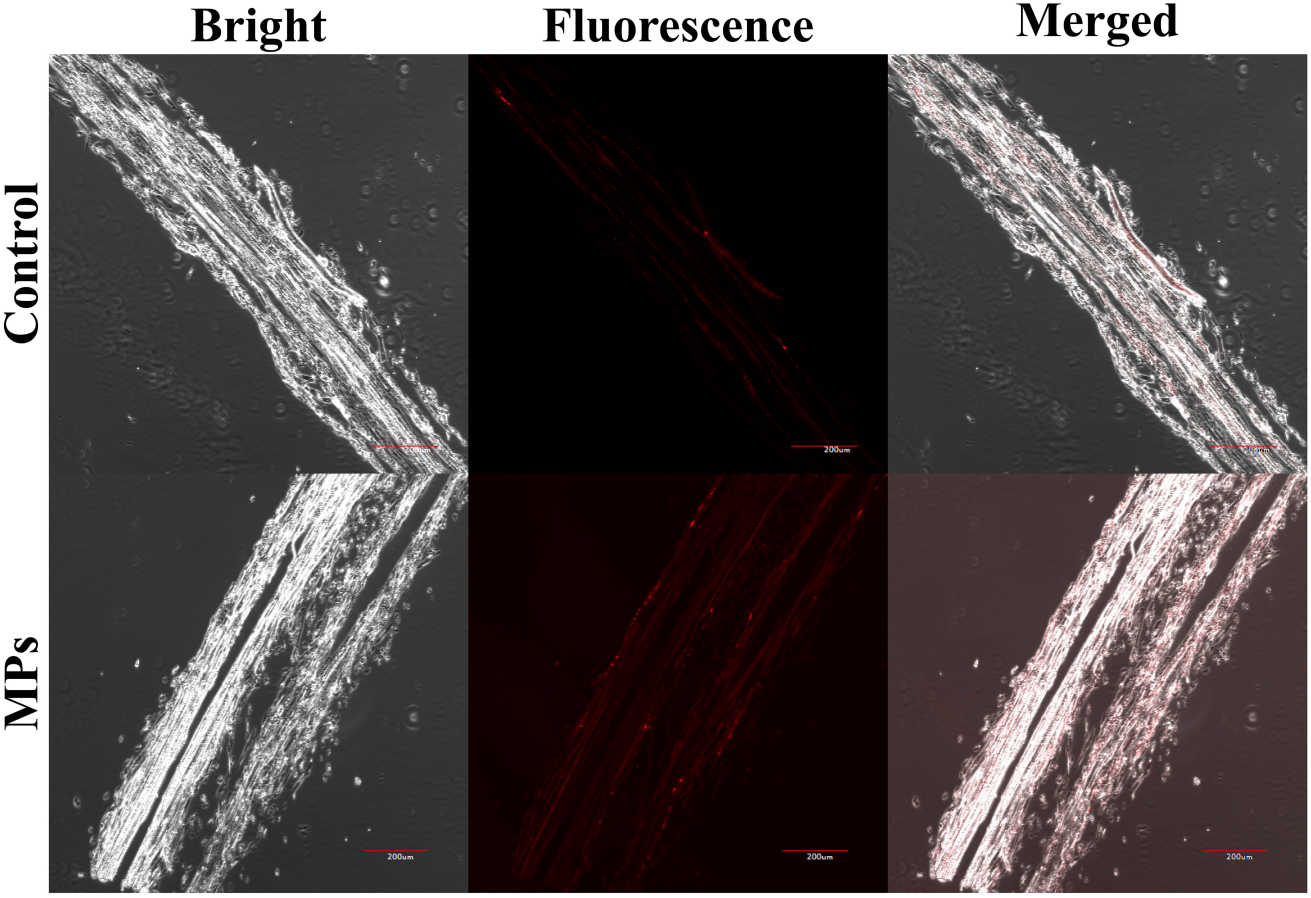
Distribution of fluorescently-labeled PMMA in highland barley roots. Scale bar, 200 μm.

Red fluorescence signals were detected at 630 nm in both the treated and control groups (**Figure 3**). The weak red fluorescent signal in the control sample was considered to be autofluorescence in the roots of highland barley. The red fluorescence signal of highland barley roots was significantly enhanced after exposure to PMMA compared to the control. This phenomenon indicates the ability of highland barley roots to absorb PMMA (**Figure 3**).

### 3.3 Effects of PS-MPs on the physiology and biochemistry of highland barley

**Figure 4:**
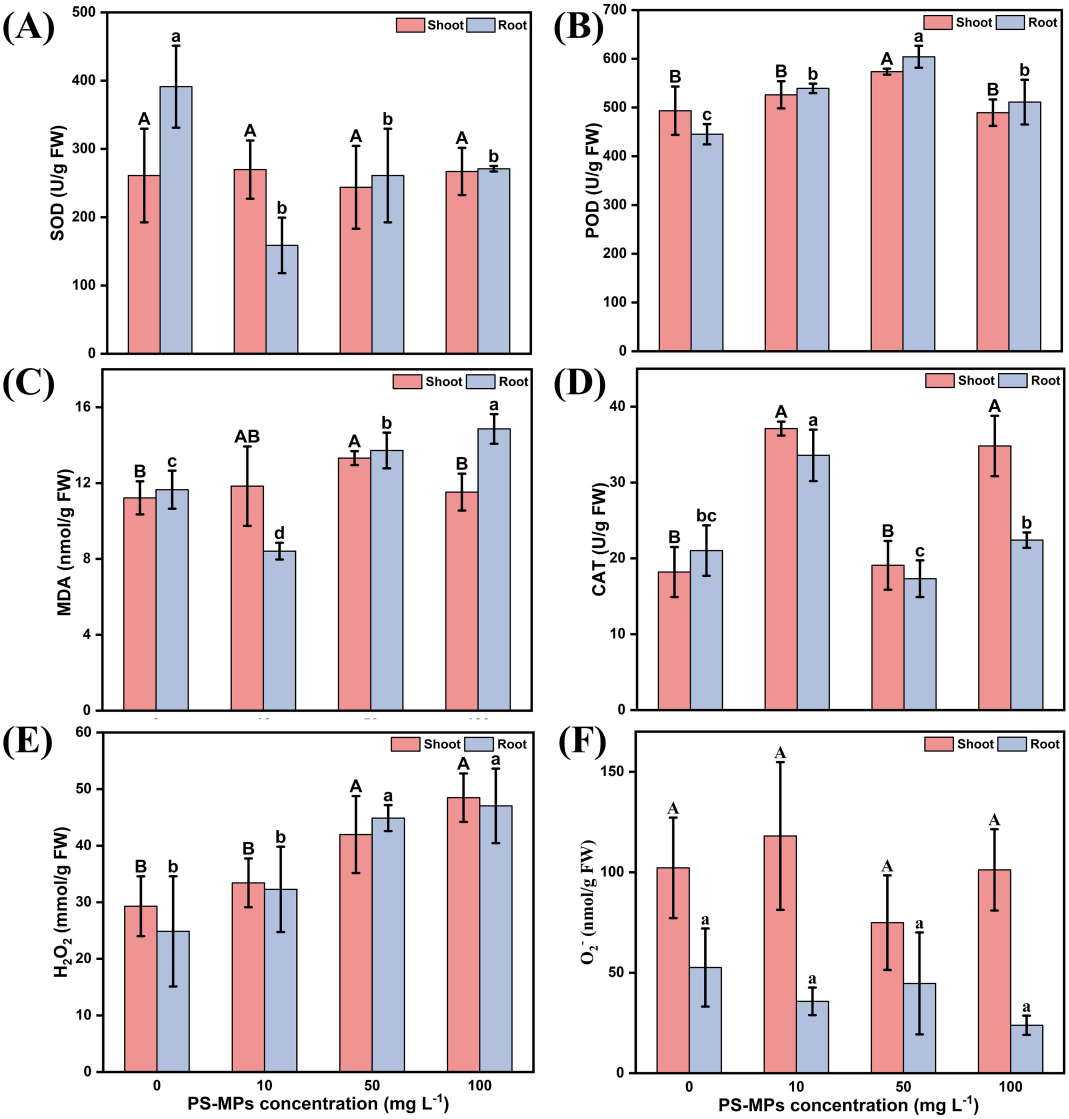
Effects of PS-MPs on the physiological indices of highland barley roots and leaves. **(A)** Superoxide Dismutase (SOD) activity **(B)** Peroxidase (POD) activity **(C)** Malondialdehyde (MDA) content **(D)** Catalase (CAT) activity **(E)** Hydrogen peroxide (H_2_O_2_) content **(F)** Superoxide Anion (O_2_^-^) Content. (Upper case letters represent differences between leaf samples and lower case letters represent differences between root samples, different letters indicate significant differences between samples) (*P* < 0.05).

The study found that varying concentrations of PS-MPs had a significant effect on the biomass and length of highland barley. Therefore, the activities of antioxidant enzymes and the MDA content in the roots and leaves of highland barley were determined. In the root samples, all concentrations of PS-MPs significantly inhibited the activity of SOD (**Figure 4A**) compared to the control, with a maximum inhibition of up to 59.4%. Additionally, they significantly increased the activity of POD (**Figure 4B**) by up to 26.3% (*P* < 0.05). The MDA content showed a decreasing and then increasing trend; specifically, low concentrations of PS-MPs significantly inhibited the activity of MDA by up to 27.8%, whereas medium and high concentrations of PS-MPs significantly increased the activity of MDA by 17.6% and 27.4%, respectively (**Figure 4C**) (*P* < 0.05). Compared with the two antioxidant enzymes, CAT showed a different trend in the highland barley roots. Low concentrations of PS-MPs significantly increased the activity of CAT, while medium and high concentrations had no significant effect (**Figure 4D**). All concentrations of MPs increased the content of hydrogen peroxide and increased with an increasing concentration (**Figure 4E**); all concentrations of PS-MPs had no significant effect on the superoxide anion in root samples (**Figure 4F**). In leaves, compared with the control group, PS-MPs had no significant effect on SOD activity in leaves (**Figure 4A**) (*P* < 0.05) but increased POD activity in leaves (**Figure 4B**) (*P* < 0.05). A similar situation was also observed for MDA (**Figure 4C**) (*P* < 0.05); CAT activity showed an increasing, then decreasing, then increasing trend., and both low and high concentrations of PS-MPs significantly increased CAT activity in leaves (**Figure 4D**) (*P* < 0.05). Similar to the root samples, all concentrations of PS-MPs increased the content of hydrogen peroxide in the leaves and increased with increasing concentrations (**Figure 4E**). All concentrations of PS-MPs had no significant effect on superoxide anions in leaf samples (**Figure 4F**) (*P* < 0.05).

### 3.4 Analysis of metabolites in highland barley roots

#### 3.4.1 Metabolite classification

**Figure 5:**
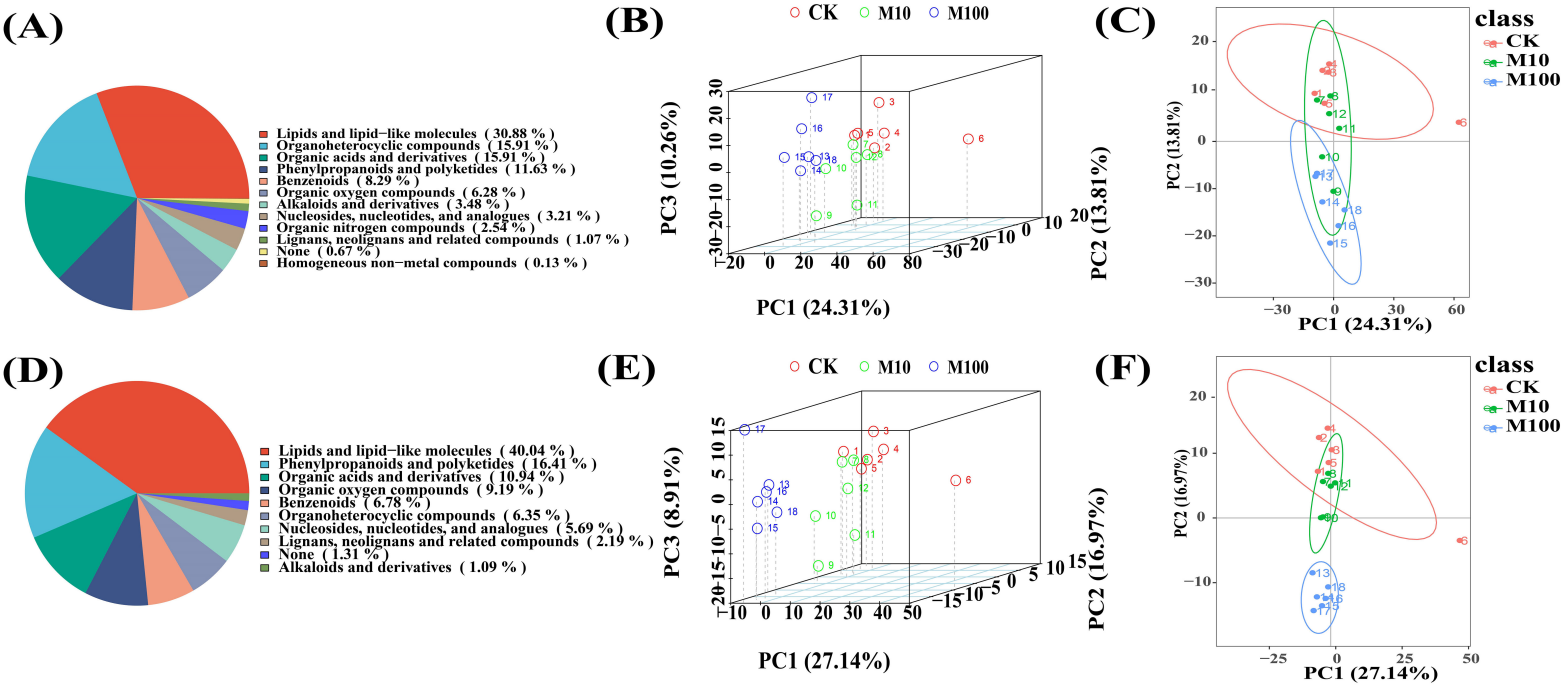
Metabolite classification and Principal Component Analysis (PCA) of samples. **(A–C)** Positive ion patterns **(D–F)** Negative ion patterns. (Horizontal and vertical coordinates PC1 and PC2 denote the scores of the first and second-ranked principal components, respectively, different-colored scatters denote samples from different experimental groupings, ellipses are 95% confidence intervals) (CK: control, M10: 10 mg/L, M00: 100 mg/L).

We used an untargeted metabolomic approach to analyze the metabolic profiles of barley roots after treatment with the control (CK), low (10 mg/L), and high (100 mg/L) concentrations of PS-MPs. In this study, two samples treated with low and high concentrations of PS-MPs were selected for metabolite extraction. This was done to reduce the saturation effect between the samples and the extraction solvents, obtain a more comprehensive metabolite profile, and improve the coverage of metabolomics analyses. The goal was to obtain optimal results from differential analysis. In positive ionmode, a total of 1,059 metabolites were detected. A total of 557 metabolites were detected in negative ionmode. Among them, in positive ionmode (**Figure 5A**), there were 231 lipids and lipid-like molecules, 119 organoheterocyclic compounds, 119 organic acids and derivatives, 87 phenylpropanoids and polyketides, and 62 benzenoids, accounting for a total of 30.88%, 15.91%, 15.91%, 11.63%, and 8.29%, respectively. In the negative ionmode (**Figure 5D**), there were 183 lipids and lipid-like molecules, 75 phenylpropanoids and polyketides, 50 organic acids and derivatives, 42 organic oxygen compounds, and 31 benzenoids, accounting for a total of 40.04%, 16.41%, 10.94%, 9.19%, and 6.78%, respectively. PCA analysis of peaks extracted from samples exposed to different doses of PS-MPs was performed to transform high-dimensional data into a low-dimensional spatial form, making it easier to visualize the data and reveal the structure and distribution more intuitively. Eighteen samples from three treatment groups were separated in this experiment, demonstrating the excellent stability of the method and high data quality. The positive ionmode is shown in **Figures 5B, C**. The negative ionmode is shown in **Figures 5E, F**.

#### 3.4.2 Metabolite annotation

**Figure 6:**
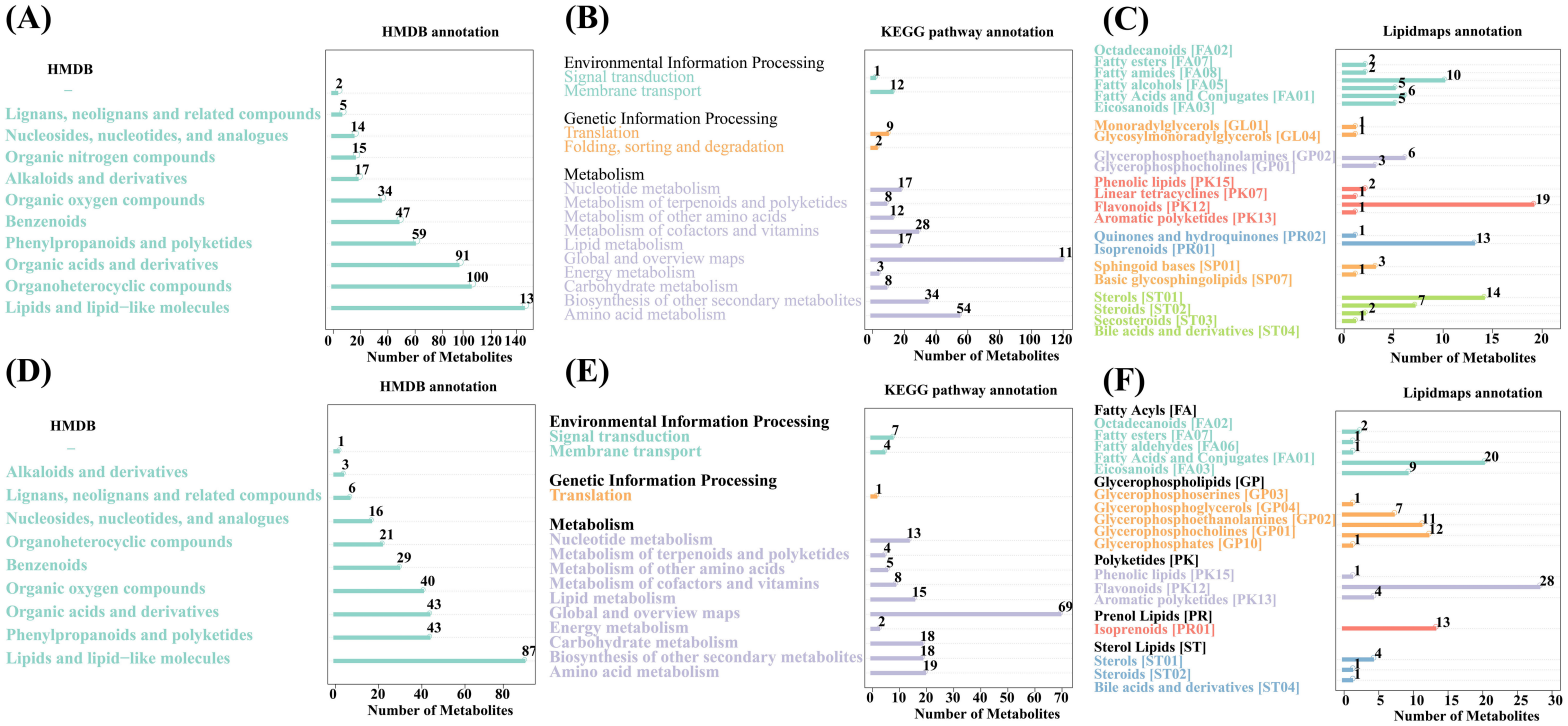
Classification of metabolic pathways against different databases. **(A–C)** Positive ion patterns **(D–F)** Negative ion patterns. The horizontal coordinates represent the number of metabolites, while the vertical coordinates represent the pathways to which they are annotated.

The identified metabolites were functionally and taxonomically annotated, and the main databases used included KEGG, HMDB, and LIPID MAPS. The identified metabolites were annotated using these databases to understand the functional properties and classification of different metabolites. We can quickly obtain the classification information of metabolites through the HMDB annotation. The annotation results of HMDB are shown in **Figure 6**. The positive ionmode contains up to 139 types of lipids and lipid-like molecules, followed by 100 types of organoheterocyclic compounds and 91 types of organic acids and derivatives (**Figure 6A**); the negative ionmode contains up to 87 types of lipids and lipid-like molecules, followed by 43 types of organoheterocyclic compounds and 43 types of organic acids and derivatives (**Figure 6D**). Pathway analysis can determine the most important biochemical metabolic pathways and signal transduction pathways involved in metabolites. The KEGG pathway annotation results are shown in **Figures 6B, E**. In positive ionmode, global and overview maps show the most metabolic pathways and signal transduction pathways in metabolism, with a total of 119; translation is the most metabolic pathway and signal transduction pathway in genetic information processing, with a total of 9; membrane transport is environmental, and there are 12 metabolic pathways and signal transduction pathways in information processing (**Figure 6B**). LIPID MAPS is a database containing biologically relevant lipid structures and annotations and it is currently the largest public lipid database in the world. The annotation results of the LIPID MAPS database are shown in **Figure 6**. In positive ionmode, fatty amides [FA08] are the most abundant metabolites in fatty acyls [FA], with 10 metabolites in total; glycerolipids [GL] containing one each of glycosylmonoradylglycerols [GL04] and monoradylglycerols [GL01]; flavonoids [PK12] are polyketides with the most metabolites in [PK], with a total of 19; sterols [ST01] contain the most metabolites in sterol lipids [ST], with a total of 14 (**Figure 6C**). In negative ionmode, fatty acids and conjugates [FA01] were the most abundant metabolite in fatty acyls [FA], with a total of 20; glycerophosphocholines [GP01] were the most abundant metabolite in glycerophospholipids [GP], with a total of 12; flavonoids [PK12] were the most abundant metabolite in polyketides [PK], with 28 in total; sterols [ST01] were the most abundant metabolite in sterol lipids [ST], with 4 in total (**Figure 6F**).

#### 3.4.3 Differential metabolite analysis

**Figure 7:**
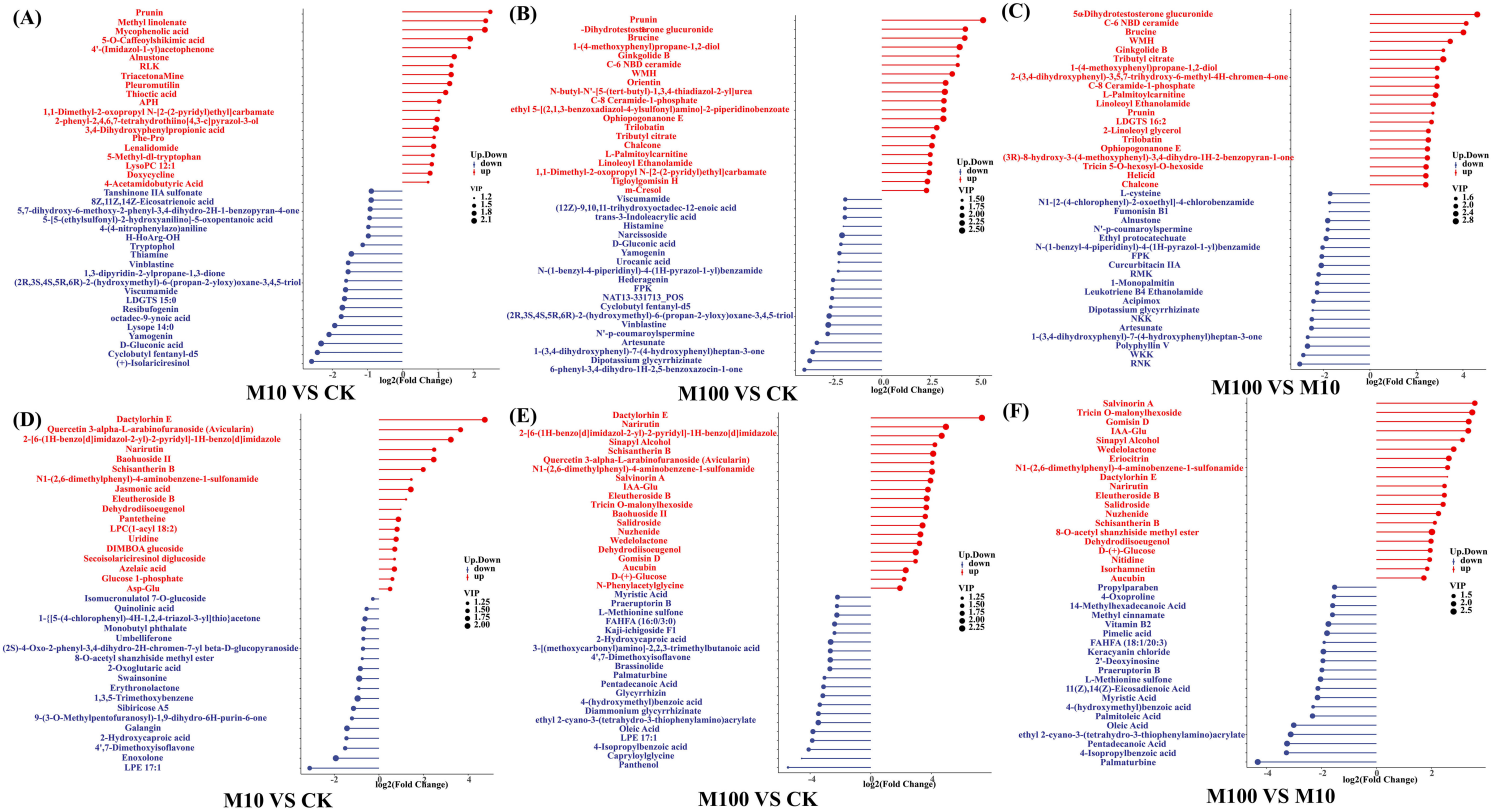
Differential metabolite matchstick diagrams. **(A–C)** Positive ionmode **(D–F)** Negative ionmode. The color of the dots represents the up and down tuning: blue represents the down tuning, and red represents the up tuning. The length of the rods represents the magnitude of log2 (Fold Change), while the size of the dots represents the magnitude of the VI value. (CK: control, M10: 10 mg/L, M00: 100 mg/L).

We compared the differential metabolites obtained by combining the differences in each group, which can indicate the upregulation of metabolites and substances with large differential changes (**Figure 7**). By analyzing and interpreting the variations of different metabolites, we can gain a deeper understanding of the regulatory mechanisms of metabolic processes, physiological functions, and biological features relevant to diseases in living organisms. This can provide important information and support for achieving research goals. In positive ionmode, treatment with low-concentration PS-MPs resulted in significant changes in 57 metabolites compared with controls (*P* < 0.05). As shown in **Figure 7A**, the contents of prunin, methyl linolenate, mycophenolic acid, 5-O-caffeoylshikimic acid, and 4’-(imidazol-1-yl)acetophenone were significantly increased by 463.9%, 415.2%, 407.0%, and 279.2%, 273.4%, respectively. The contents of (+)-isolariciresinol, cyclobutyl fentanyl-d5, D-gluconic acid, yamagenin, and lysoPE 14:0 were significantly reduced by 83.2%, 81.2%, 79.8%, 76.3%, and 73.5%, respectively. As shown in **Figure 7E**, treatment with high concentrations of PS-MPs resulted in significant changes in 163 metabolites compared to the control (*P* < 0.05). The contents of prunin, 5α-dihydrotestosterone glucuronide, brucine, 1-(4-methoxyphenyl)propane-1,2-diol, and ginkgolide B were significantly increased by 3581.8%, 1856.3%, 1815.7%, 1499.3%, and 1403.2%, respectively. 6-Phenyl-3,4-dihydro-1H-2,5-benzoxazocin-1-one, dipotassium glycyrrhizinate, 1-(3,4-dihydroxyphenyl)-7-(4-hydroxyphenyl)heptan-3-one, artesunate, and N’-p-coumaroylspermine were the five metabolites with the most significant content reductions, which decreased by 93.7%, 92.3%, 91.5%, 90.1%, and 95.5%, respectively (*P* < 0.05). Compared with the low-concentration treatment, the high-concentration treatment resulted in significant changes in 156 metabolites; 96 metabolites were significantly increased, and 60 metabolites were significantly decreased (*P* < 0.05) (**Figure 7C**). In the negative ionmode, treatment with low-concentration PS-MPs resulted in significant changes in 36 metabolites compared with the control (*P* < 0.05). As shown in **Figure 7D**, the contents of Dactylorhiza E, Quercetin 3-alpha-L-arabinofuranoside (Avicularin), 2-[6-(1H-benzo[d]imidazol-2-yl)-2-pyridyl]-1H-benzo[d]imidazole, Narirutin, and Baohuoside II were significantly increased by 2584.2%, 1164.2%, 834.2%, 456.5%, and 449.2%, respectively. LPE 17:1, enoxolone, 4’,7-dimethoxyisoflavone, 2-hydroxycaproic acid, and galangin were the five metabolites with the most significant content reduction, which were reduced by 88.39%, 73.8%, 65.0%, 63.6%, and 63.0%, respectively. As shown in **Figure 7E**, treatment with high concentrations of PS-MPs resulted in significant changes in 119 metabolites compared to the control (*P* < 0.05). The contents of dactylorhin E, narirutin, 2-[6-(1H-benzo[d]imidazol-2-yl)-2-pyridyl]-1H-benzo[d]imidazole, sinapyl alcohol, and schisantherin B were significantly increased by 15932.5%, 2994.3%, 2451.6%, 1769.9%, and 1627.5%, respectively. Panthenol, capryloylglycine, 4-isopropylbenzoic acid, LPE 17:1, and oleic acid were the five metabolites with the most significant reductions, which were reduced by 97.7%, 95.7%, 94.2%, 93.1%, and 92.9%, respectively. Compared with the low-concentration treatment, the high-concentration treatment resulted in significant changes in 122 metabolites, with 44 metabolites significantly increased and 78 metabolites significantly decreased (*P* < 0.05) (**Figure 7F**).

Hierarchical clustering analysis (HCA) was performed on all the differential metabolites between each comparison pair obtained (Chen et al., 2015) (**Supplementary Figures S1**, **S2**). In positive ionmode, 257 metabolites were significantly changed (*P* < 0.05) in all samples exposed to PS-MPs (**Supplementary Figure S1**). Prunin, 1,1-dimethyl-2-oxopropyl, N-[2-(2-pyridyl)ethyl]carbamate, nicotinate ribonucleoside, and 4-acetamidobutyric acid metabolite contents increased significantly with an increasing concentration of PS-MPs (*P* < 0.05). The contents of vinblastine and (2R,3S,4S,5R,6R)-2-(hydroxymethyl)-6-(propan-2-yloxy)oxane-3,4,5-triol metabolites were instead significantly decreased (*P* < 0.05) with increasing concentrations of PS-MPs (*P* < 0.05). In addition, some metabolites responded differently after exposure to different concentrations of PS-MPs. The contents of the metabolites alnustone, 3,4-dihydroxyphenylpropionic acid, 2-phenyl-2,4,6,7-tetrahydrothiino[4,3-c]pyrazol-3-ol, and N4-phenethylmorpholine-4-carbothioamide increased significantly at low concentrations, but the contents decreased significantly with increasing concentrations of PS-MPs (*P* < 0.05). The contents of (+)-catechin and 5-[5-(ethylsulfonyl)-2-hydroxyanilino]-5-oxopentanoic acid metabolites decreased significantly after exposure to low concentrations of PS-MPs, but increased with increasing concentrations of PS-MPs and significantly increased (*P* < 0.05). In negative ionmode, 177 metabolites were significantly changed (*P* < 0.05) in all samples exposed to PS-MPs (**Supplementary Figure S2**). Dactylorhin E, Schisantherin B, Dehydrodiisoeugenol, Narirutin, Eleutheroside B, N1-(2,6-dimethylphenyl)-4-aminobenzene-1-sulfonamide, Secoisolariciresinol diglucoside, and Glucose 1-phosphate metabolite contents all increased significantly with the concentration of PS-MPs (*P* < 0.05). The contents of 2-hydroxycaproic acid, 9-(3-O-methylpentofuranosyl)-1,9-dihydro-6H-purin-6-one, and 4’,7-dimethoxyisoflavone metabolites significantly decreased with increasing concentrations of PS-MPs (*P* < 0.05). The content of azelaic acid metabolites was significantly increased after exposure to low concentrations of PS-MPs but significantly decreased with increasing PS-MP concentrations (*P* < 0.05). Conversely, the content of the 8-O-acetyl shanzhiside methyl ester metabolite was significantly decreased after exposure to low concentrations of PS-MPs, but it increased significantly with increasing concentrations of PS-MPs (*P* < 0.05).

#### 3.4.4 Venn diagram analysis and correlation analysis of metabolites

**Figure 8:**
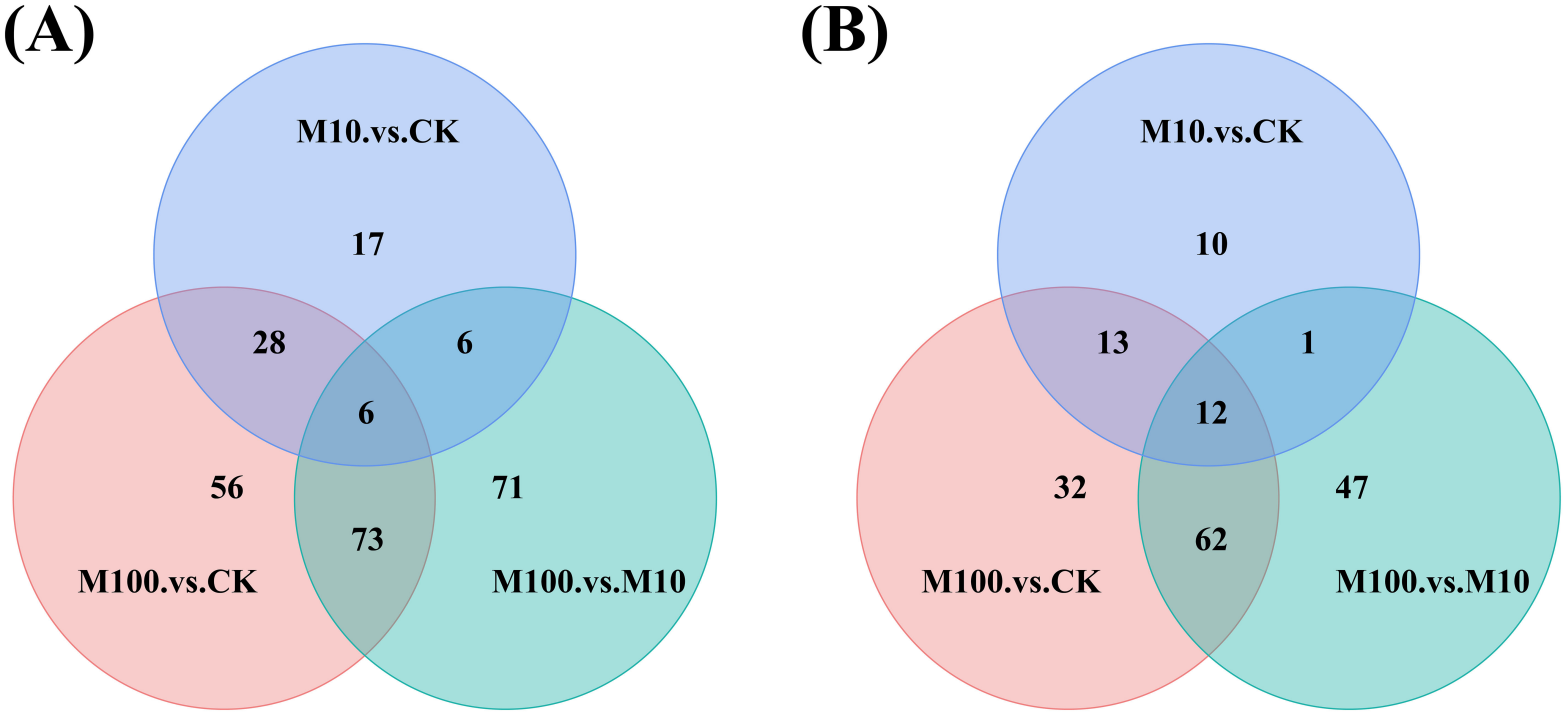
Venn diagrams of differentially expressed metabolites. **(A)** Positive ionmode **(B)** Negative ionmode. (CK: control, M10: 10 mg/L, M00: 100 mg/L).

Through a Venn diagram, the differential metabolites in multiple comparison groups can be visually compared, displaying both overlapping and unique differential metabolites between different groups (**Figure 8**). In positive-ion mode (**Figure 8A**), there were 17 unique differential metabolites in the M10 and CK samples compared to all combinations. Additionally, there were 56 unique differential metabolites in the M100 and CK samples and 71 unique differential metabolites in the M100 and M10 samples. There were 6 differential metabolites in all combinations. There were 34 common differential metabolites in the M100, CK, and M10, CK groups of samples; 12 common differential metabolites in the M100, M10, and M10, CK groups of samples; and 79 common differential metabolites in the M100, M10, and M100, CK groups of samples. In negative ionmode (**Figure 8B**), compared with all combinations, there were 10 unique differential metabolites in the M10 and CK samples, 32 unique differential metabolites in the M100 and CK samples, and 47 unique differential metabolites in the M100 and 10 samples. There were 12 different metabolites in all combinations. There were 25 common differential metabolites in the M100, CK, and M10, CK groups of samples; 13 common differential metabolites in the M100, M10, and M10, CK groups of samples; and 74 common differential metabolites in the M100, M10, and M100, CK groups of samples.

**Figure 9:**
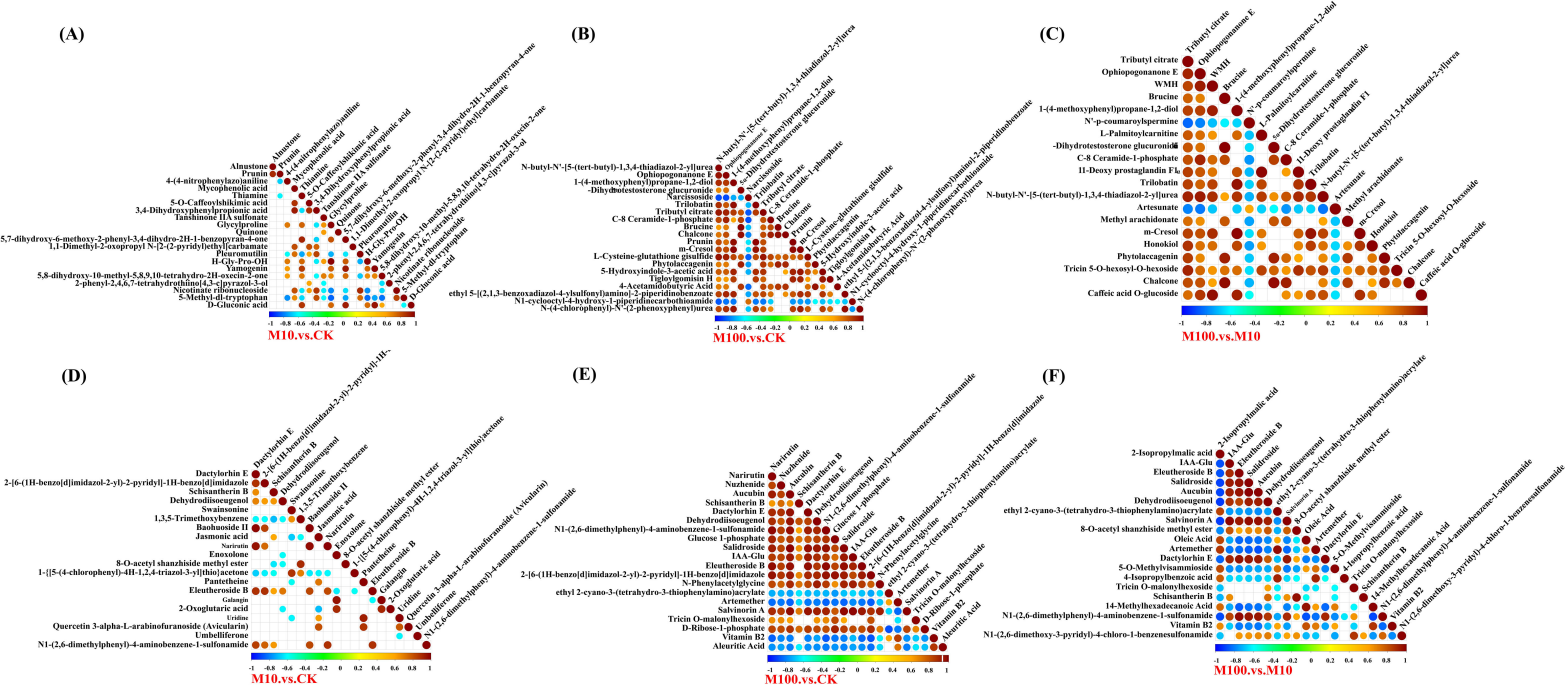
Correlation plots of differentially expressed metabolites. **(A–C)** Positive ion patterns **(D–F)** Negative ion patterns. The highest correlation is 1, which indicates a perfect positive correlation (red); the lowest correlation is -1, which indicates a perfect negative correlation (blue); and the part without color indicates a *P*-value > 0.05. The figure shows the correlation of the top 20 differential metabolites, sorted from smallest to largest *P*-value. (CK: control, M10: 10 mg/L, M00: 100 mg/L).

Statistical test of significance for correlations among different metabolites (Rao et al., 2016). In the positive ion mode, as shown in **Figures 9A–C**, compared to the control, H-Gly-Pro-OH and glycylproline showed the highest positive correlation at low concentrations, with a correlation coefficient of 1. On the other hand, 5-methyl-dl-tryptophan was negatively correlated with glycylproline, with a correlation coefficient of 0.84. Compared to the control, brucine and 5α-dihydrotestosterone glucuronide showed the highest positive correlation at high concentrations, with a correlation coefficient of 1. N-butyl-N’-[5-(tert-butyl)-1,3,4-thiadiazol-2-yl]urea and narcissoside showed the highest negative correlation, with a correlation coefficient of 0.88. Compared to low concentrations, brucine, and 5α-dihydrotestosterone glucuronide had the highest positive correlation at high concentrations, with a correlation coefficient of 0.99. On the other hand, tributyl citrate and N’-p-coumaroylspermine had the highest negative correlation, with a correlation coefficient of 0.84. In the negative ion mode, as shown in **Figures 9D–F**, compared to the control, baohuoside II and dactylorhin E showed the highest positive correlation with a correlation coefficient of 0.98. Schisantherin B and 1,3,5-trimethoxybenzene showed the highest negative correlation with a correlation coefficient of 0.70. Compared to the control, aucubin and salidroside showed the highest positive correlation at high concentrations, with a correlation coefficient of 1. Vitamin B2 and 2-[6-(1H-benzo[d]imidazol-2-yl)-2-pyridyl]-1H-benzo[d]imidazole showed the highest negative correlation, with a correlation coefficient of 0.87. Compared to low concentrations, salidroside and aucubin had the highest positive correlation at high concentrations, with a correlation coefficient of 1. Dehydrodiisoeugenol and 2-isopropylmalic acid had the most negative correlation, with a correlation coefficient of 0.91.

### 3.5 Affected biological pathways in highland barley roots

**Figure 10:**
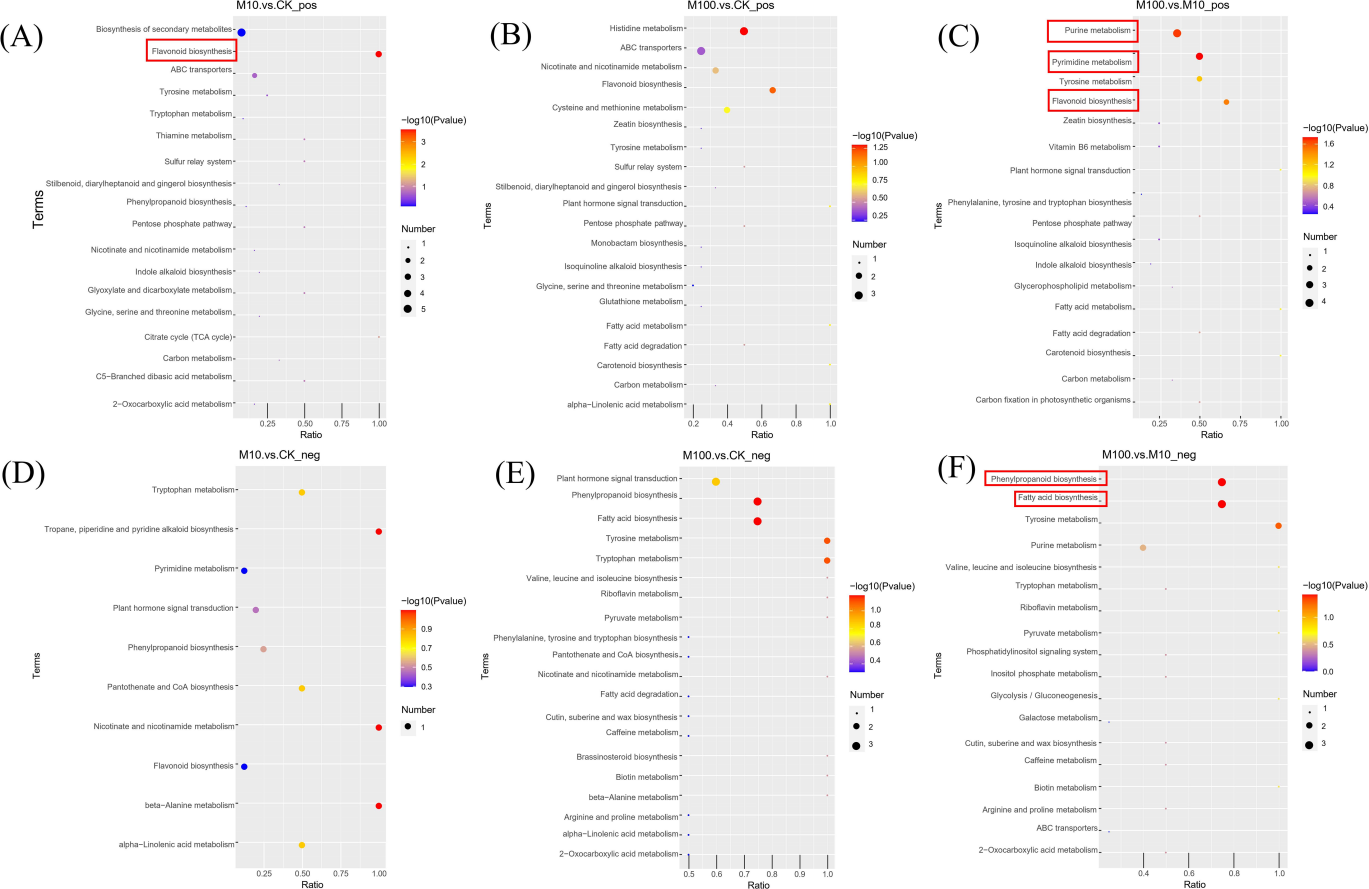
Bubble plots of KEGG-enriched pathways. **(A–C)** Positive ionmode **(D–F)** Negative ionmode. The horizontal coordinate in the graph represents the ratio of the number of differential metabolites in the corresponding metabolic pathway to the total number of metabolites identified in the pathway. A larger value indicates a higher degree of differential metabolite enrichment in the pathway. The color of the dots represents the *P*-value of the hypergeometric test; the smaller the value, the more reliable and statistically significant the test is. The size of the dots represents the number of differential metabolites in the corresponding pathway. The larger the value, the more differential metabolites there are in the pathway. (CK: control, M10: 10 mg/L, M00: 100 mg/L).

By studying the affected metabolic pathways, it is possible to gain a more comprehensive understanding of the adaptive and physiological characteristics of plateau barley under the stress of PS-MPs. The results of the biological pathway analysis showed that, as shown in **Figure 10**, exposure to different doses of PS-MPs significantly disturbed the metabolic pathways of flavonoid biosynthesis, pyrimidine metabolism, purine metabolism, fatty acid biosynthesis, and phenylpropanoid biosynthesis in highland barley roots. Specifically, in the positive ionmode, compared with the control, upregulation of Prunin and caffeoyl shikimic acid metabolites and downregulation of (+)-gallocatechin metabolites are key factors affecting the flavonoid biosynthesis metabolic pathway in root samples exposed to low concentrations of PS-MPs (**Supplementary Figure S3**). The downregulation of deoxycytidine, 5-methylcytosine, and thymine metabolites was the main factor affecting the metabolic pathway of pyrimidine metabolism in root samples at high concentrations of PS-MPs compared with low concentrations (**Supplementary Figure S4**). In addition, the downregulation of hypoxanthine and deoxycytidine metabolites and the upregulation of AMP metabolites significantly affected the metabolic pathways of purine metabolism (**Supplementary Figure S5**). In negative ionmode, compared with low concentrations, the downregulation of tetradecanoic acid, hexadecenoic, and octadecenoic acid metabolites in high-concentration root samples was the key factor affecting the metabolic pathway of fatty acid biosynthesis (**Supplementary Figure S6**); the upregulation of sinapyl alcohol and syringin metabolites and sinapic acid metabolites significantly affected the metabolic pathways of phenylpropanoid biosynthesis (**Supplementary Figure S7**).

## 4 Discussion

### 4.1 Effects of different concentrations of microplastics on the growth of highland barley

The ecological pollution caused by PS-MPs has received extensive attention worldwide (Maity and Pramanick, 2020). The presence of PS-MPs may affect the structure and function of microbial communities in the oceans and soils, negatively impacting biodegradation and ecological processes. More importantly, PS-MPs can be accidentally ingested or absorbed by marine and terrestrial organisms and then passed on to humans through the food chain. Long-term ingestion may pose a potential threat to human health (Stabnikova et al., 2021; Yin et al., 2021). In addition, PS-MPs can have a positive or negative effect on seed germination, plant morphology, and growth physiology, either directly or indirectly. For example, Wu et al (Wu et al., 2020a). exposed rice to a solution of MPs at a concentration of 500 mg/L. They found that the aboveground biomass of rice was reduced by 40.3%. Under agro-cultivation conditions (250 mg/kg), the aboveground biomass of rice was reduced by 25.9%. Urbina et al (Urbina et al., 2020). exposed maize to a 100 mg/L solution of MPs and found a 50% reduction in height and biomass. According to research, there is a significant correlation between biomass and crop yield. A decrease in biomass is usually accompanied by a decrease in yield. Given that highland barley is a major food crop in the highlands of China, the extent to which it is affected by microplastic pollution is of even greater concern.

In this study, it was found that a low concentration (10 mg/L) and a medium concentration (50 mg/L) of PS-MPs increased the biomass and length of the highland barley leaves and roots, while a high concentration (100 mg/L) of PS-MPs significantly reduced the biomass and length of both the highland barley leaves and roots (**Figure 2**). Different concentrations of MPs had different effects on the growth of plants. Studies have found that low concentrations of PS-MPs (10 mg/L) can significantly increase the root length of rice (Zhang et al., 2021), while Jiang et al (Jiang et al., 2019). have shown that high concentrations of MPs (100 mg/L) can inhibit crop growth. Interestingly, Bouaicha et al (Bouaicha et al., 2022). found that a high concentration (100 mg/L) of PE-MPs could significantly increase the barley root biomass(30.2%). Changes in plant biomass may be related to plant species and types of MPs. In addition, Zhang et al (Zhang et al., 2022a). found that PS-NPs with different particle sizes had a significant effect on the biomass of plant stems. We believe that an appropriate concentration and particle size of MPs can significantly promote plant growth; this requires further exploration. MPs can enter through cracks at the initiation site of lateral roots in plants (Li et al., 2020b). The reduction in biomass and length may be related to the disruption of nutrient transport caused by the uptake of PS-MPs in the plant roots. It has been shown that PS-MPs cause damage to the structure of plant root hairs and may lead to morphological abnormalities in root hairs and alterations in secretions, which can affect nutrient uptake (Ren et al., 2022). In addition, some studies have found that PS-MPs, after entering the root tissues, interact with the cell membrane and may cause a breakage of the cell membrane, increasing permeability (Zhang et al., 2022b). This may also lead to a decrease in the nutrient uptake capacity of the plant root system. Therefore, we suggest that the decline in nutrient transport capacity and nutrient uptake is responsible for the reduction in highland barley biomass and length.

Through research, we found that there was a weak red fluorescent signal in the roots of highland barley in the control group (**Figure 3**); this was considered to be the spontaneous fluorescent signal of the plant’s roots (Li et al., 2020b; Zhou et al., 2021). However, a more significant fluorescence signal appeared in the root samples treated with PS-MPs. By comparing the intensity of fluorescence, it can be concluded that the roots of highland barley can absorb PS-MPs. Li et al (Li et al., 2021a). observed the same phenomenon in barley root samples. The possibility of transporting PS-NPs in rice roots was also confirmed (Zhou et al., 2021). More seriously, the accumulation of MPs was found in the stem, leaf, calyx, and fruit of cucumber (Li et al., 2021b). MPs were also found to accumulate in the roots of rice seedlings and transfer them to the stems and leaves (Liu et al., 2022). MPs in fruits can be transferred into the human body, posing potential health risks.

In this paper, the effects of MPs on the growth of highland barley were revealed for the first time, and the absorption of MPs in the roots was observed. The results of this study provide strong support for further research on the harm of MPs. In future studies, special attention should be paid to the absorption of MPs in the roots, stems, and leaves of plants to prevent MPs from reaching the human body through the enrichment of the food chain.

### 4.2 Effects of different concentrations of microplastics on the physiology and biochemistry of highland barley

Typically, exposure to adverse environmental conditions triggers oxidative stress in plants, which is reflected in the formation of reactive oxygen species (ROS) in plant cells (Alscher et al., 1997). While plant cells are protected from oxidative damage caused by reactive oxygen species (Liu et al., 2015), plants can regulate a series of antioxidant enzymes to scavenge ROS (Wiegand and Pflugmacher, 2005; Nel et al., 2006; Wang et al., 2010). O_2_^−^ and H_2_O_2_ are key ROS, and their content can reflect the level of ROS stress in plants (Zhang et al., 2021). POD, SOD, and CAT are the main enzymes in the plant antioxidant system, and their activity levels can reflect the degree of adverse effects on plants. SOD can catalyze the conversion of O_2_^−^ into H_2_O_2_ and O_2_ and is the primary substance for scavenging free radicals in living organisms (Wojtaszek, 1997; Lin and Kao, 2000; Martinez et al., 2001). CAT and POD are enzymes that scavenge H_2_O_2_. The three compounds maintain a steady-state level of free radicals in plants through synergistic action, preventing the changes in plant physiology and biochemistry caused by free radicals (Weckx and Clijsters, 1996).

This study found that in highland barley roots (**Figure 4**), all concentrations of PS-MPs significantly inhibited SOD activity and increased POD activity, while CAT activity was significantly increased at the 10 mg/L concentration (*P* < 0.05) but not significantly changed at the 50 mg/L and 100 mg/L concentrations. In the roots of highland barley, the decrease in SOD activity may be related to the accumulation of ROS in the roots. At the same time, the increase in POD activity and the change in CAT activity in roots reflected the metabolic state of plants and their adaptability to the external environment. A similar phenomenon was found in the study of antioxidant enzyme activity of polystyrene microplastics on rice seeds by Zhang et al (Zhang et al., 2021). In leaves, PS-MPs had no significant effect on SOD activity, while POD activity showed a tendency to increase and then decrease. The changes in CAT activity were complex, showing a trend of increasing, then decreasing, and then increasing. POD is involved in redox reactions in plants and plays an important role in antioxidant defense. The observed first increase and then decrease in activity may reflect the plant’s response to initial environmental stimuli, such as oxidative stress. The first increase in activity may be an adaptive response of the plant to stress, enhancing antioxidant defenses to scavenge excess reactive oxygen species. However, when the stress is sustained or excessive, it may lead to inhibition of POD, resulting in a decrease in its activity (Zhang et al., 2024). The initial increase in CAT activity may be a response to an increase in reactive oxygen species, helping to catabolize hydrogen peroxide and reduce oxidative stress. Subsequent decreases in activity may reflect inhibition or depletion of enzyme activity or the effects of regulatory mechanisms. However, as the plant adapts to environmental changes or recovers, a renewed increase in CAT activity may indicate that the plant’s antioxidant defenses are effectively restored and re-established to adapt to the new growing conditions (Lian et al., 2022; Zhang et al., 2022b; Sun et al., 2023). In addition, the decrease in CAT activity at high concentrations may be due to the production of H_2_O_2_ when SOD is induced, which is decomposed under the action of CAT. As a result, the activity of CAT is greatly reduced due to the large consumption of it. Enhancement of antioxidant enzyme activity helps eliminate excess ROS (Lai and Luo, 2019).

This study found that different concentrations of PS-MPs caused varying levels of damage to the antioxidant system in different parts of highland barley (**Figure 4**). This is in agreement with the findings of Lu et al (Lu et al., 2023), who found that PS-MPs inhibit plant growth and induce oxidative stress. Li et al (Li et al., 2021a). analysed antioxidant enzyme activities in different parts of rice and found that treatment of PS-MPs at 2 g/mL significantly increased the activities of dehydroascorbate reductase (DHAR) and glutathione reductase (GR) in the roots, which in turn promoted the ascorbate-glutathione (AsA-GSH) cycle. However, it significantly decreased the cell wall peroxidase (cwPOX) activity in the root. In the leaves, MPs only increased the DHAR activity, while other antioxidant enzyme activities were not affected by MPs. In addition, Wang, Z.S. et al (Wang et al., 2022b). found that the presence of PS-NPs (2 g/L)significantly reduced the activities of superoxide dismutase, ascorbate peroxidase, and catalase in chloroplasts and the activities of ascorbate peroxidase and catalase in mitochondria under low-temperature conditions (2°C). Therefore, we suggest that culture conditions may also be an important factor contributing to changes in plant antioxidant enzymes.

Interestingly, the H_2_O_2_ content in all samples increased with increasing concentrations, especially at 50 mg/L and 100 mg/L, while there was no significant effect on O_2_^−^. As one of the most abundant reactive oxygen species (ROS) in cells, H_2_O_2_ is a key signaling molecule for plant growth and development and resistance to stress and, it plays an important role in the response of plants to environmental stress (He et al., 2021). The increase in H_2_O_2_ content also indicated changes in ROS in plants. In addition, ROS may be formed outside the scavenging ability of the antioxidant system, resulting in decreased membrane activity and thus changes in MDA content (Wu et al., 2020a).

MDA is the main product of lipid peroxidation. The MDA content can be used as an important indicator to reflect the degree of lipid peroxidation in plant cell membranes. The higher the content, the greater the damage to the biofilm. In the present study, a decreasing and then increasing trend of MDA was found in the root samples. Specifically, low concentrations decreased the MDA content, while medium and high concentrations significantly increased it. In contrast, the MDA content in leaves showed an increasing and then decreasing trend. Importantly, MDA levels in roots and leaves were higher in all treatment groups than in the control, except for the low-concentration root samples and high-concentration shoot samples. This suggests that PS-MPs caused a greater degree of disruption to the biofilm of plateau barley. Increased MDA content has also been observed in some studies, including cucumber (Li et al., 2021b; Zhang et al., 2023), Cicer arietinum L (Dey et al., 2023), corn (Zhang et al., 2022a), and rice (Ma et al., 2022).

By monitoring and understanding changes in plant enzyme activity, we can better assess plant adaptability to adverse environmental conditions and ecosystem health. A transient increase in enzyme activity is a regulatory response that protects the body from external stress and toxicity, while a decrease in activity indicates that the threshold of self-regulation ability has been exceeded, and the enzyme has been damaged (Liao et al., 2019). For example, increasing the activity of POD and CAT enzymes can help plants remove excess oxidizing substances and reduce oxidative damage, thereby enhancing the plant’s ability to adapt to environmental stress (Borges et al., 2023). This study is the first to report the effects of different concentrations of MPs on the physiology and biozchemistry of highland barley. The results show that plants respond to the stress of MPs through diverse modes, including the regulation of ROS and the activities of antioxidant enzymes.

### 4.3 Effects of different concentrations of microplastics on the metabolism of highland barley

We investigated the metabolic profiles of highland barley roots exposed to different concentrations of PS-MPs. The root system is the main site of water and nutrient uptake in plants, the part of the plant initially exposed to microplastics, and it produces specific metabolites in response to environmental stresses. Therefore, when studying the effects of microplastics on plants, changes in the metabolism of the root system can often more directly reflect the response of plants to environmental stress. Metabolomics can reveal the metabolic regulatory mechanisms behind observed changes in plant metabolites (Yun et al., 2020). Studies have shown that the metabolome is influenced by endogenous and environmental molecules (Tuteja and Sopory, 2008), and plants can synthesize and accumulate a variety of specialized metabolites, including biologically active alkaloids and terpenoids (Shoji and Yuan, 2021). To date, studies have reported the effects of MPs on the plant metabolome, including rice (Wu et al., 2020a), barley, cucumber, tomato (Bouaicha et al., 2022), corn (Zhang et al., 2022a), and lettuce (Wang et al., 2023a), etc. Highland barley is the smallest staple grain and the largest coarse grain in China (Guo et al., 2020). However, no study has reported the effect of MPs on the metabolome of seedlings.

In this study, metabolome analysis of highland barley seedling root samples treated with different concentrations of PS-MPs revealed significant differences in 257 metabolites in the positive-ion mode. Among these metabolites, the content of four increased with PS-MPs, including prunin, 1,1-dimethyl-2-oxopropyl, N-[2-(2-pyridyl)ethyl]carbamate, nicotinate ribonucleoside, and 4-acetamidobutyric acid (**Supplementary Figure S1**). Wei et al (Wei et al., 2021). discovered that prunin is a significant factor causing discoloration in the Phyllostachys violascens cultivar. Céliz et al (Céliz et al., 2013). pointed out that prunin has a strong antioxidant capacity and can effectively prevent oxidative damage. Additionally, Matsui and Ashihara found that nicotinic ribonucleoside is an important metabolite in plants that can act as a coenzyme for oxidoreductases (Matsui and Ashihara, 2008). Therefore, we believe that to protect highland barley root cells from oxidative damage caused by reactive oxygen species, a series of enzymes, such as prunin and nicotinamide ribonucleotide, are upregulated to scavenge ROS. In contrast, the content of vinblastine and (2R,3S,4S,5R,6R)-2-(hydroxymethyl)-6-(propan-2-yloxy)oxane-3,4,5-triol metabolites decreased significantly with increasing concentrations of PS-MPs (*P* < 0.05). Studies have reported that changes in vinblastine content can reflect the stress response of plants under environmental stress (Liu et al., 2017). In addition, some metabolites responded differently after exposure to different concentrations of PS-MPs. For example, the contents of the metabolites alnustone, 3,4-dihydroxyphenylpropionic acid, 2-phenyl-2,4,6,7-tetrahydrothiino[4,3-c]pyrazol-3-ol, and N4-phenethylmorpholine-4-carbothioamide increased significantly at low concentrations, but the contents decreased significantly with increasing concentrations of PS-MPs (*P* < 0.05). The contents of (+)-catechin and 5-[5-(ethylsulfonyl)-2-hydroxyanilino]-5-oxopentanoic acid metabolites decreased significantly after exposure to low concentrations of PS-MPs but increased with increasing concentrations of PS-MPs and significantly increased (*P* < 0.05). Changes in metabolite contents revealed stress responses in highland barley root samples under different concentrations of PS-MPs. In the negative ionmode, 177 metabolites were significantly changed (*P* < 0.05). Among them, Dactylorhin E, Schisantherin B, Dehydrodiisoeugenol, Narirutin, Eleutheroside B, N1-(2,6-dimethylphenyl)-4-aminobenzene-1-sulfonamide, Secoisolariciresinol diglucoside, and Glucose 1-phosphate metabolites increased significantly with the concentrations of PS-MPs (*P* < 0.05). At present, a large number of studies have reported on schisantherin B (Ma et al., 2019; Olas, 2023), dehydrodiisoeugenol (Godínez-Chaparro et al., 2022), narirutin (Kim and Lim, 2020; Mitra et al., 2022), and secosolariciresinol diglucoside (Huang et al., 2018). Such metabolites have high antioxidant activity and can prevent oxidative stress, which provides support for the results of this study. Furthermore, in the context of abiotic stress, plant metabolites can directly act as elicitors and signals (such as amino acids) for plant adaptation mechanisms (Hildebrandt et al., 2015). The contents of 2-hydroxycaproic acid, 9-(3-O-methylpentofuranosyl)-1,9-dihydro-6H-purin-6-one, and 4’,7-dimethoxyisoflavone metabolites significantly decreased with increasing concentrations of PS-MPs (*P* < 0.05), indicating that the root metabolism was adversely affected. In this paper, a metabolomic system was used to reveal, for the first time, the metabolic response mechanism of highland barley in response to MPs stress. The increased content of metabolites with high antioxidant activity is an important pathway for plants to respond to MPs stress.

### 4.4 Metabolic function enrichment of highland barley in response to microplastics

Changes in metabolic pathways may interfere with the crop’s antioxidant defense systems, including energy metabolism and anabolism (Wu et al., 2020a). The results of the biological pathway analysis (**Figure 10**) showed that the metabolic pathways of flavonoid biosynthesis, pyrimidine metabolism, purine metabolism, fatty acid biosynthesis, and phenylpropanoid biosynthesis in highland barley roots were significantly disrupted after exposure to different doses of PS-MPs (**Supplementary Figures S3**–**S7**). Flavonoid biosynthesis is an important class of secondary metabolites widely present in plants, which contribute to plant growth and development (Liu et al., 2021). In addition, plant growth is also associated with the phenylpropanoid biosynthesis metabolism (Muro-Villanueva et al., 2019). Nucleotide biosynthesis and metabolism are also critical for plant growth and development (Stasolla et al., 2003). Notably, under abiotic stress conditions, plants exhibit increased synthesis of polyphenols such as phenolic acids and flavonoids, which help them cope with environmental constraints (Sharma et al., 2019). Fatty acids and lipids are major and essential components of all plant cells, not only providing structural integrity and energy for various metabolic processes but also functioning as signal transduction mediators (Lim et al., 2017). The phenylpropanoid biosynthetic pathway is activated under abiotic stress conditions, leading to the accumulation of various phenolic compounds, such as metabolites with the potential to scavenge harmful reactive oxygen species (Sharma et al., 2019). Significant changes in these metabolic pathways revealed the stress response of highland barley roots in response to interference from PS-MPs. Changes in metabolite content in metabolic pathways may reduce the nutritional quality or yield of highland barley (Wu et al., 2022), so MPs contamination should arouse our attention. Overall, an untargeted metabolomic analysis qualitatively and quantitatively identified metabolic pathways affected by PS-MPs in the highland barley roots (Zhao et al., 2022). This study is the first to report changes in the metabolic pathways of highland barley under the stress of MPs. Among them, the metabolic pathway of pyrimidine metabolism was discovered for the first time in plants stressed by MPs.

## 5 Conclusions

Studies have shown that microplastics have adverse effects on plant growth. As an important grain crop in the high-altitude area of China, the safety of highland barley should be given more attention. In this paper, we used metabolomics technology for the first time to study the effects of different concentrations of microplastics on physiological indicators and metabolites in highland barley and revealed the response mechanism of barley seedlings to PS-MPs. The results showed that low (10 mg/L) and medium (50 mg/L) concentrations of PS-MPs increased aboveground biomass and root length, while high (100 mg/L) concentrations decreased aboveground biomass and root length. The uptake of MPs by the roots may interrupt nutrient transport, resulting in a decrease in biomass and length. Indicators of oxidative stress (antioxidant enzymes, ROS, MDA) showed that oxidative stress was activated in highland barley under PS-MPs stress. Non-targeted metabolomics showed an increase in antioxidant-active metabolites (including populin, ribonucleoside nicotinic acid, pentosidine B, and dehydrodiisoeugenol) in the root system. The PS-MPs mainly interfere with the normal metabolism of flavonoid biosynthesis, pyrimidine metabolism, purine metabolism, fatty acid biosynthesis, and phenylpropanoid biosynthesis. These pathways are closely related to the mechanisms of PS-MPs’ duress in highland barley. The present study provides new perspectives for understanding the potential impacts of microplastics on crops and contributes to a more comprehensive assessment of their hazards to plant production systems. In future research, the effects of different types and shapes of microplastics on crops should be explored. Varieties that are more resistant to microplastic stress should also be bred. Additionally, environmental monitoring and management should be strengthened, and appropriate strategies should be developed to reduce the impact of microplastics on agroecosystems.

## Data Availability

The raw data supporting the conclusions of this article will be made available by the authors, without undue reservation.

## Supplementary material

Supplementary Figure 1 Heat map of total differential metabolite clustering of positive ions. The colors change from red to blue, representing the content changing from high to low. The red font represents that the content of metabolites has been increasing from control to M100, the yellow font represents that the content of metabolites has been decreasing from control to M100, the green font represents that the content of metabolites has been increasing and then decreasing from control to M10, and then to M100, and the blue font represents that the content of metabolites has been decreasing and then increasing from control to M10, and then to M100. (CK: control, M10: 10 mg/L, M00: 100 mg/L).

Supplementary Figure 2 Heat map of total differential metabolite clustering of negative ions. The color changes from red to blue, representing the content changing from high to low. The red font represents that the content of metabolites has been increasing from control to M100, yellow font represents that the content of metabolites has been decreasing from control to M100, green font represents that the content of metabolites has been increasing and then decreasing from control to M10 then to M100, and blue font represents that the content of metabolites has been decreasing and then increasing from control to M10 then to M100. (CK: control, M10: 10 mg/L, M00: 100 mg/L).

Supplementary Figure 3 Metabolic pathways of flavonoid biosynthesis. Note: Circles represent metabolites, with green solid circles marking annotated metabolites, red circles indicating up-regulated differential metabolites, blue circles indicating down-regulated differential metabolites, and yellow circles indicating the inclusion of both up- and down-regulated metabolites.

Supplementary Figure 4 Metabolic pathways of pyrimidine metabolism. Note: Circles represent metabolites, with green solid circles marking annotated metabolites, red circles indicating up-regulated differential metabolites, blue circles indicating down-regulated differential metabolites, and yellow circles indicating the inclusion of both up- and down-regulated metabolites.

Supplementary Figure 5 Metabolic pathways of purine metabolism. Note: Circles represent metabolites, with green solid circles marking annotated metabolites, red circles indicating up-regulated differential metabolites, blue circles indicating down-regulated differential metabolites, and yellow circles indicating the inclusion of both up- and down-regulated metabolites.

Supplementary Figure 6 Metabolic pathways for fatty acid biosynthesis. Note: Circles represent metabolites, with green solid circles marking annotated metabolites, red circles indicating up-regulated differential metabolites, blue circles indicating down-regulated differential metabolites, and yellow circles indicating the inclusion of both up- and down-regulated metabolites.

Supplementary Figure 7 Metabolic pathways of phenylpropanoid biosynthesis. Note: Circles represent metabolites, with green solid circles marking annotated metabolites, red circles indicating up-regulated differential metabolites, blue circles indicating down-regulated differential metabolites, and yellow circles indicating the inclusion of both up- and down-regulated metabolites.
