# Supplementary figures and images for: Effects of polystyrene microplastics on the growth and metabolism of highland barley seedlings based on LC-MS
Source: Front Plant Sci. 2024 Dec 17;15:1477605. doi: 10.3389/fpls.2024.1477605 (PMC11685026; doi:10.3389/fpls.2024.1477605)

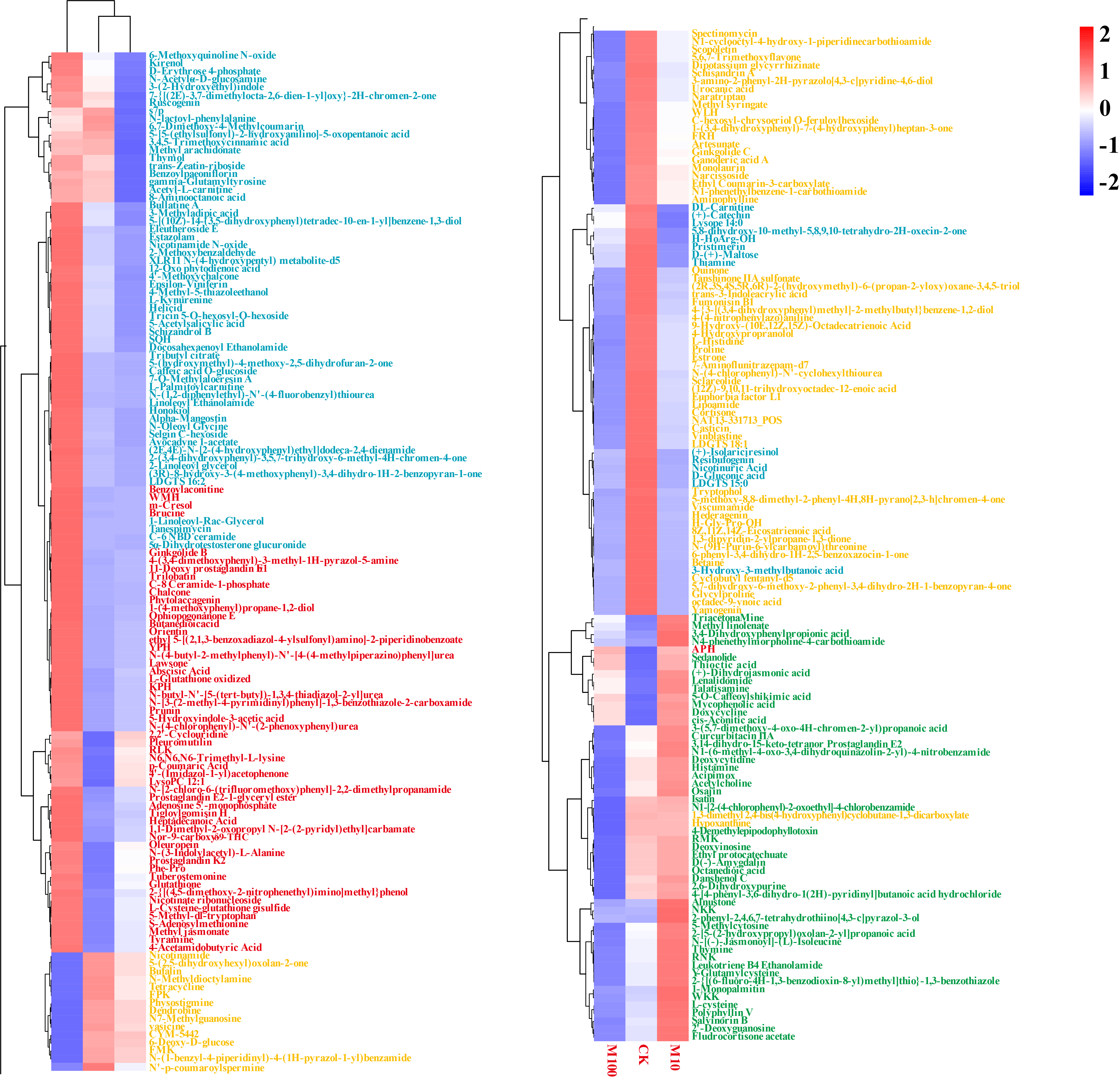

Supplement: Supplementary Figure 1 — Heat map of total differential metabolite clustering of positive ions. The colors change from red to blue, representing the content changing from high to low. The red font represents that the content of metabolites has been increasing from control to M100, the yellow font represents that the content of metabolites has been decreasing from control to M100, the green font represents that the content of metabolites has been increasing and then decreasing from control to M10, and then to M100, and the blue font represents that the content of metabolites has been decreasing and then increasing from control to M10, and then to M100. (CK: control, M10: 10 mg/L, M00: 100 mg/L). [file Image1.tif]

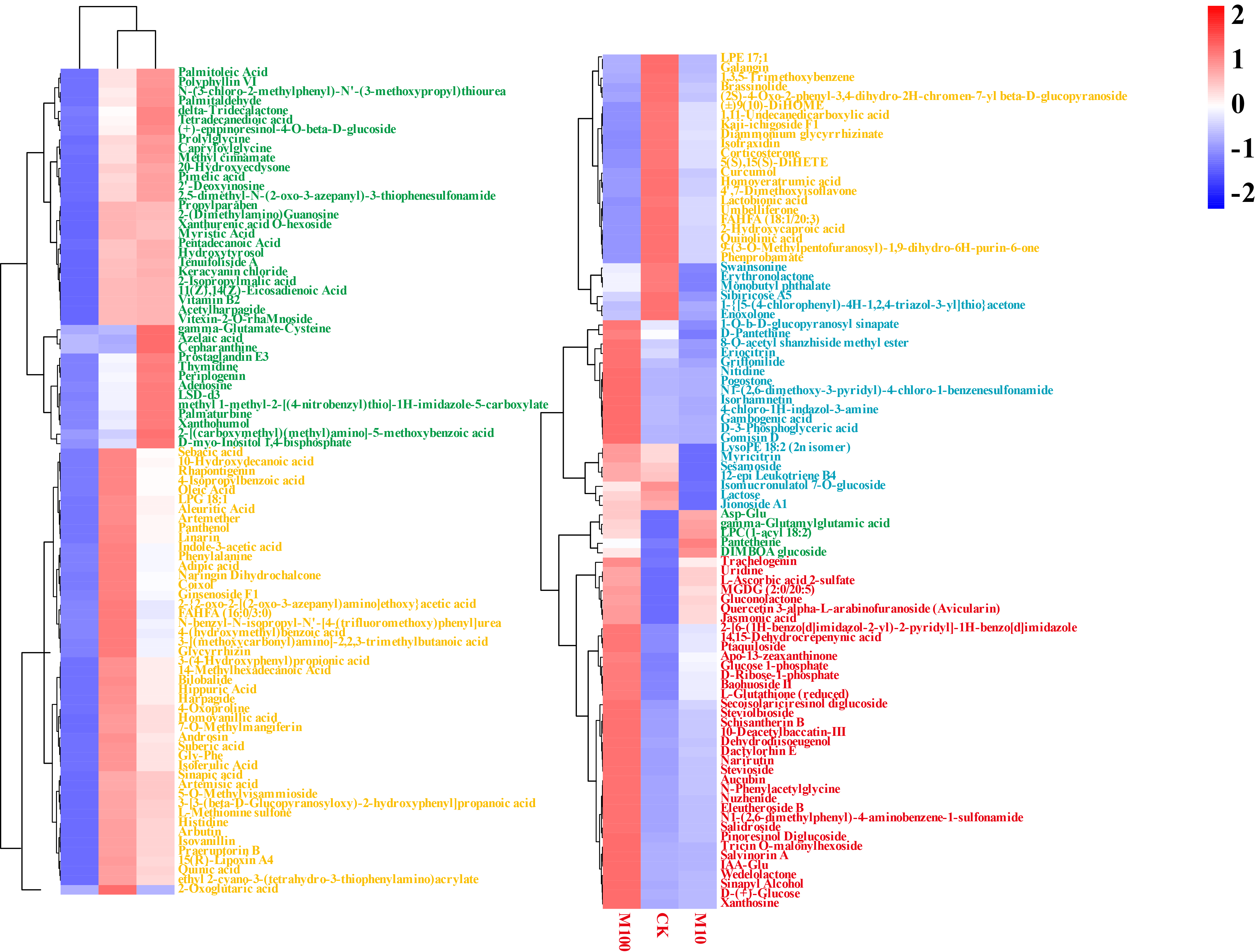

Supplement: Supplementary Figure 2 — Heat map of total differential metabolite clustering of negative ions. The color changes from red to blue, representing the content changing from high to low. The red font represents that the content of metabolites has been increasing from control to M100, yellow font represents that the content of metabolites has been decreasing from control to M100, green font represents that the content of metabolites has been increasing and then decreasing from control to M10 then to M100, and blue font represents that the content of metabolites has been decreasing and then increasing from control to M10 then to M100. (CK: control, M10: 10 mg/L, M00: 100 mg/L). [file Image2.tif]

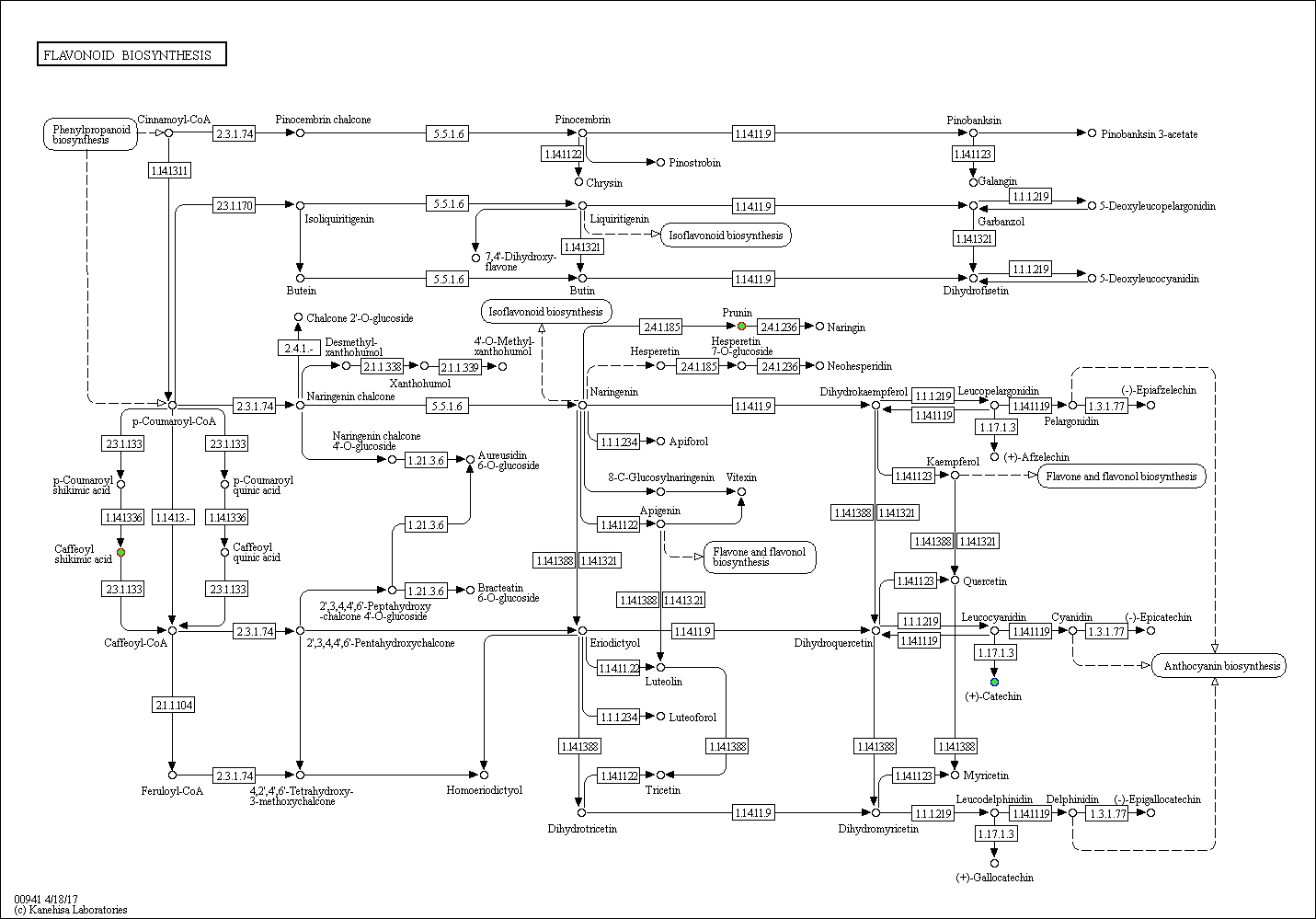

Supplement: Supplementary Figure 3 — Metabolic pathways of flavonoid biosynthesis. Note: Circles represent metabolites, with green solid circles marking annotated metabolites, red circles indicating up-regulated differential metabolites, blue circles indicating down-regulated differential metabolites, and yellow circles indicating the inclusion of both up- and down-regulated metabolites. [file Image3.png]

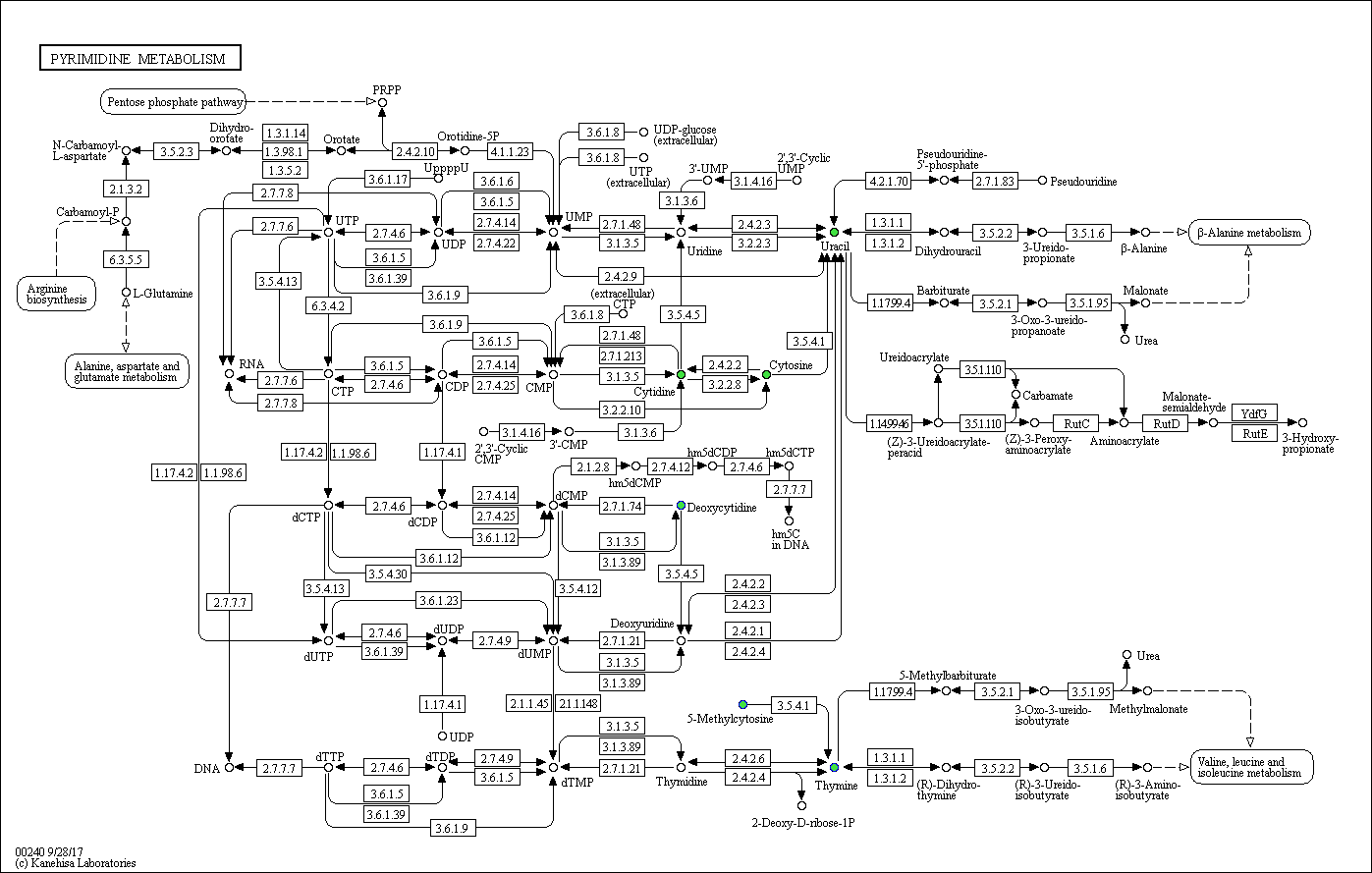

Supplement: Supplementary Figure 4 — Metabolic pathways of pyrimidine metabolism. Note: Circles represent metabolites, with green solid circles marking annotated metabolites, red circles indicating up-regulated differential metabolites, blue circles indicating down-regulated differential metabolites, and yellow circles indicating the inclusion of both up- and down-regulated metabolites. [file Image4.png]

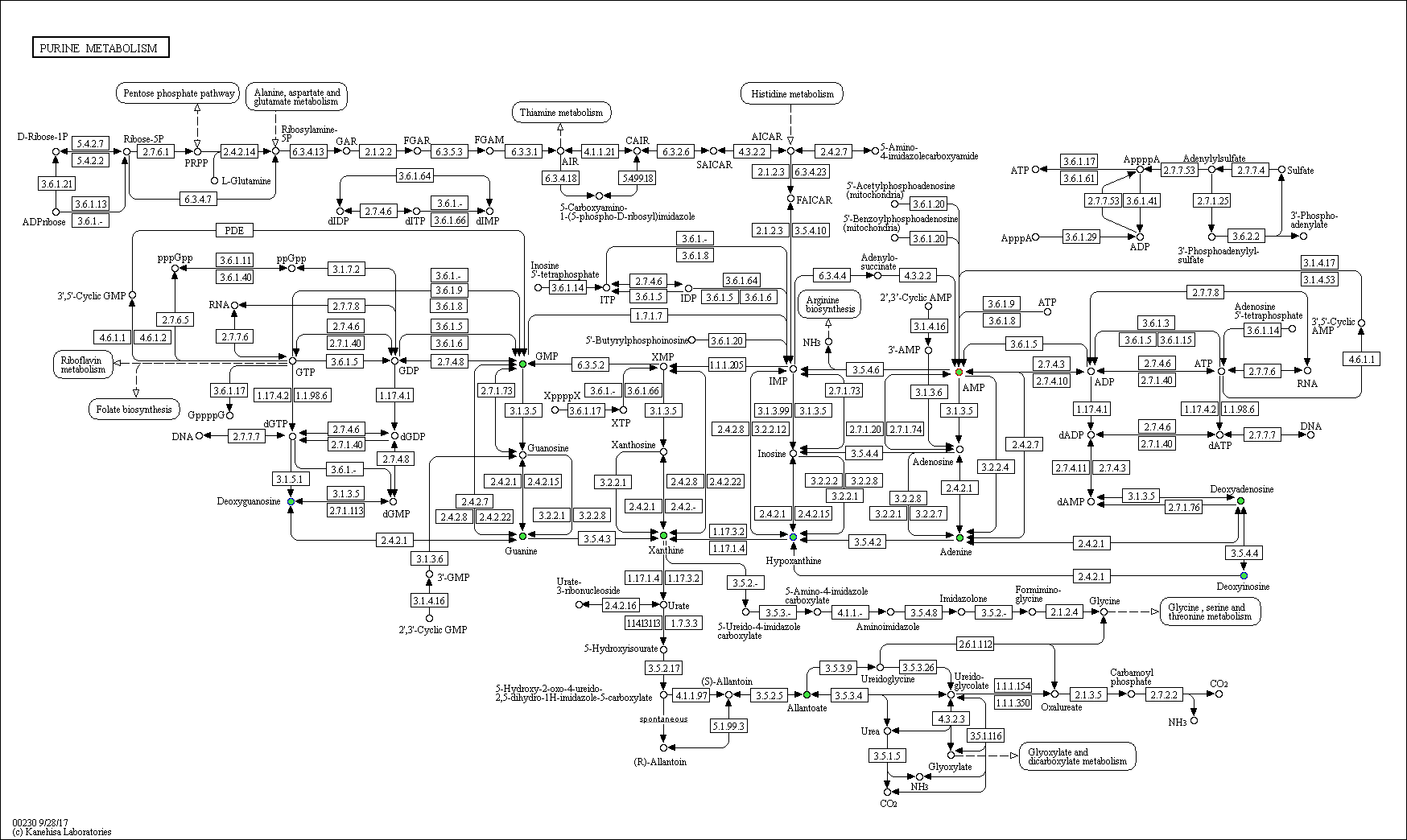

Supplement: Supplementary Figure 5 — Metabolic pathways of purine metabolism. Note: Circles represent metabolites, with green solid circles marking annotated metabolites, red circles indicating up-regulated differential metabolites, blue circles indicating down-regulated differential metabolites, and yellow circles indicating the inclusion of both up- and down-regulated metabolites. [file Image5.png]

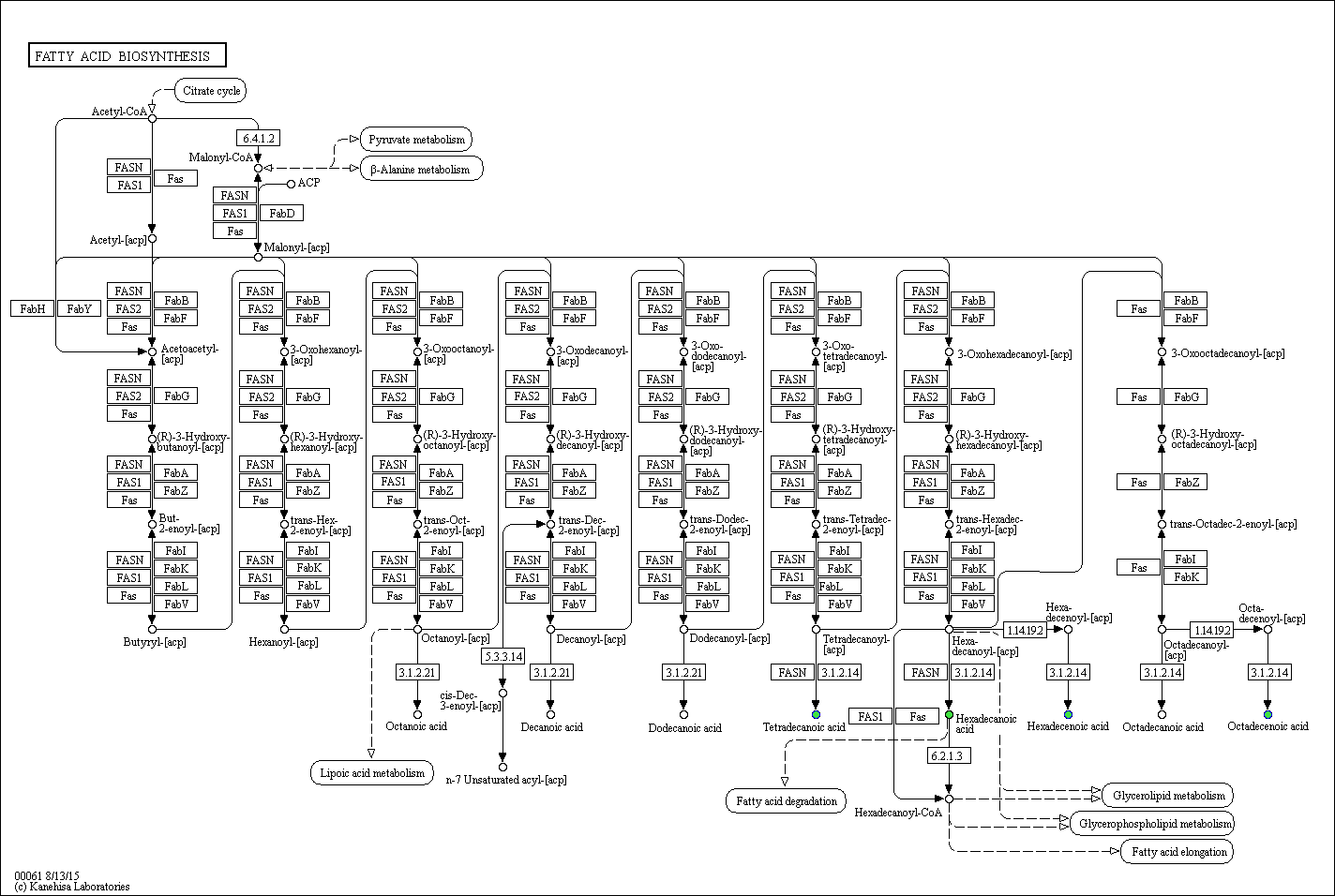

Supplement: Supplementary Figure 6 — Metabolic pathways for fatty acid biosynthesis. Note: Circles represent metabolites, with green solid circles marking annotated metabolites, red circles indicating up-regulated differential metabolites, blue circles indicating down-regulated differential metabolites, and yellow circles indicating the inclusion of both up- and down-regulated metabolites. [file Image6.png]

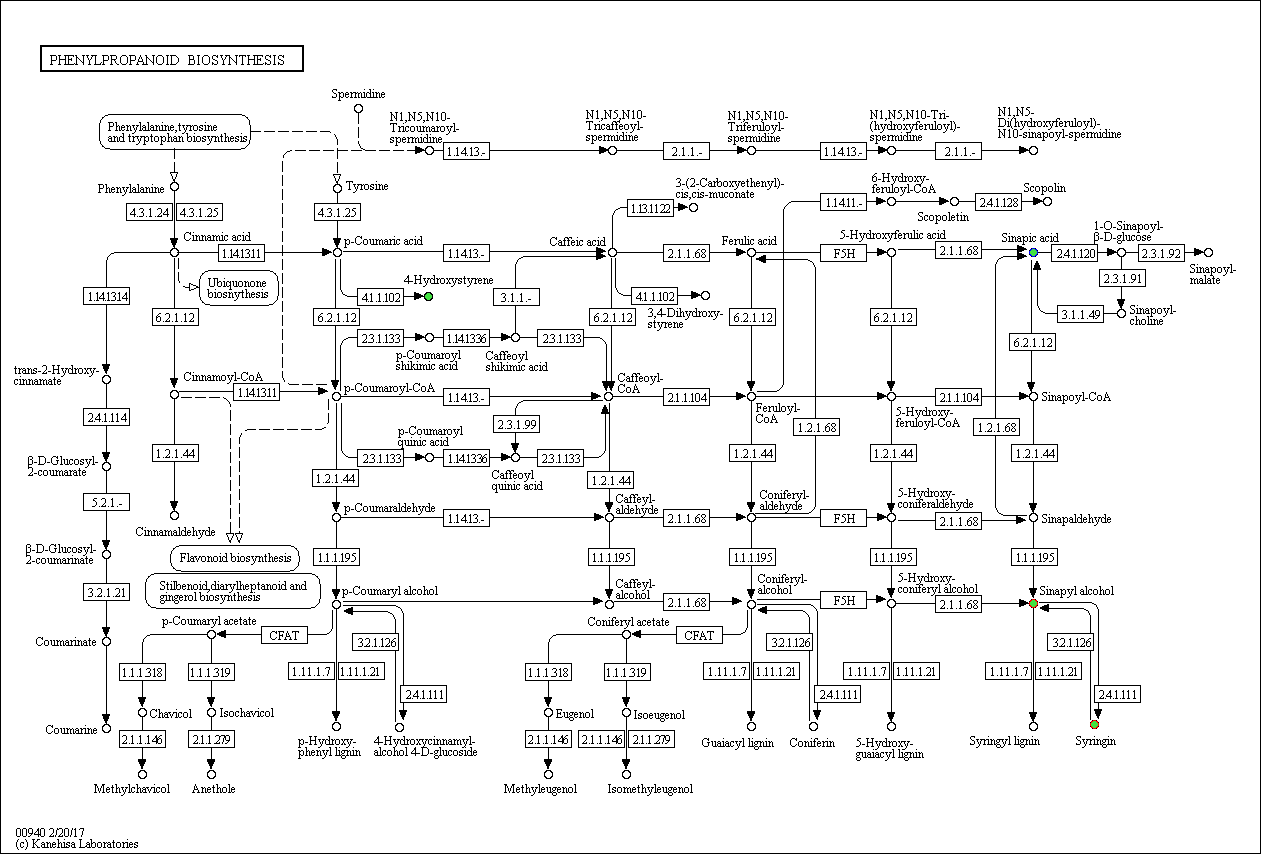

Supplement: Supplementary Figure 7 — Metabolic pathways of phenylpropanoid biosynthesis. Note: Circles represent metabolites, with green solid circles marking annotated metabolites, red circles indicating up-regulated differential metabolites, blue circles indicating down-regulated differential metabolites, and yellow circles indicating the inclusion of both up- and down-regulated metabolites. [file Image7.png]
